# Loss of TET2 sulfhydration results in destabilization of TET2 and down-regulation of the immune response in melanoma

**DOI:** 10.1016/j.apsb.2026.04.010

**Published:** 2026-04-19

**Authors:** Di Wang, Weiwei Deng, Yang Xie, Ansheng Xie, Yuan Chang, Shuheng Li, Yishan Chen, Leiqiong Gao, Yan Zhang, Zhiying Yu, Bocheng Wang, Yingping Xu, Haijing Wu, Yan Ding, Bin Yang, Lian Zhang, Yunsheng Liang

**Affiliations:** a Hunan Key Laboratory of Medical Epigenomics & Department of Dermatology, The Second Xiangya Hospital of Central South University, Changsha 410011, China; b Dermatology Hospital of Southern Medical University, Guangzhou 510091, China; c Department of Dermatology, The 3rd Affiliated Hospital of Sun Yat-sen University, Guangzhou 510630, China; d Department of Neurology, Nanfang Hospital, Southern Medical University, Guangzhou 510515, China; e Institute of Immunological Innovation and Translation, Chongqing Medical University, Chongqing 400016, China; f Department of Dermatology, Hainan Provincial Hospital of Skin Disease, Haikou 570206, China

**Keywords:** Melanoma, TET2 protein, Active sulfur, *S*-sulfhydration, DNA hydroxymethylation, Epigenetics, Immunotherapy, PD-1

## Abstract

Loss of TET2 protein leads to reduced DNA 5-hydroxymethylation (5-hmC), a key epigenetic alteration in melanoma, yet the regulatory mechanism governing TET2 stability remains unclear. Here, TET2 sulfhydration was analyzed in clinical melanoma samples, with sulfhydration sites identified by mass spectrometry, and the effects of active sulfur on TET2 stability, catalytic activity, and global 5-hmC assessed in melanoma cells. TET2 sulfhydration was significantly depleted in melanoma and further reduced in advanced stages. The cysteine-rich zinc finger domain at C1186/1202 was identified as the key sulfhydration site, which is essential for maintaining TET2 stability and enzymatic activity. Active sulfur restored TET2 sulfhydration, up-regulated TET2 protein, and reprogrammed global DNA hydroxymethylation. Notably, active sulfur synergized with anti-PD-1 therapy by enhancing IFN-*γ*-induced Th1-type chemokine and MHC-I expression in melanoma cells, thereby boosting CD8^+^ T-cell-mediated immune responses. These findings demonstrate that TET2 sulfhydration at C1186/1202 is a crucial post-translational modification for TET2 stability and function, playing a key role in epigenetic regulation and antitumor immunity, and highlight the immunoadjuvant potential of reactive sulfur species in melanoma immunotherapy.

## Graphical abstract

TET2 sulfhydration at C1186/1202 maintains its stability and epigenetic function. The restoration of TET2 sulfhydration by active sulfur enhances antitumor immunity and synergizes with anti-PD-1 therapy in melanoma.

**Figure:**
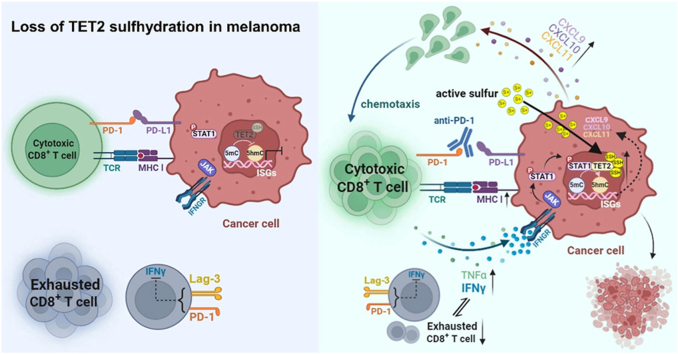

## 1 Introduction

A reduction in ten-eleven translocation (TET) proteins, especially TET2, is an important epigenetic alteration in melanoma^1^^,^^2^. TET enzymes catalyze the hydroxylation of 5-methylcytosine (5-mC) into 5-hydroxymethylcytosine (5-hmC), erasing methylation marks crucial for epigenetic regulation^3^. Dysfunctional TET2 is linked to abnormal DNA hydroxymethylation and an unfavorable prognosis in patients with melanoma^4^. Studies have shown that reintroducing TET2 can restore its expression and inhibit tumor growth by reinstating 5-hmC in melanoma cells^4^, ^5^, ^6^. Maintaining TET2 function is crucial for suppressing malignancy.

Beyond its epigenetic role in tumor cells, TET2 has emerged as a pivotal regulator of antitumor immunity. TET2-mediated DNA demethylation modulates the transcription of immune-related genes, influencing antigen presentation, chemokine secretion, and cytokine responses. In immune cells, TET2 controls the differentiation and effector function of CD8^+^ T cells, the stability of regulatory T cells, and the pro-inflammatory capacity of myeloid cells, thereby shaping tumor–immune interactions^7^, ^8^, ^9^, ^10^, ^11^. For instance, deficiency of TET2 within tumor-associated macrophages promotes their polarization toward an immunosuppressive phenotype, whereas TET2 loss in T cells compromises both memory development and cytotoxic activity^12^. In melanoma, reduced TET2 activity has been associated with impaired recruitment of effector T cells^2^, suggesting a direct link between TET2 function and immune checkpoint responsiveness.

However, the regulation of TET2 protein expression still needs further investigation. Emerging evidence identifies vitamin C, oxygen homeostasis, and posttranslational modifications (PTMs) networks as major determinants of TET2 regulation at both expression and enzymatic function^5^^,^^13^, ^14^, ^15^, ^16^. *S*-Sulfhydration, a recently discovered PTM, is implicated in cellular functions and metabolic processes. Furthermore, active sulfur, which acts as a sulfhydryl donor represented by sodium hydrosulfide (NaHS), significantly contributes to the development of tumors and autoimmune diseases *via* regulation of protein *S*-sulfhydration at cysteine residues^10^^,^^17^, ^18^, ^19^. Interestingly, the zinc-finger structure of TET2 contains a cysteine-rich region that could be a potential target for sulfhydration.

TET2 regulation by sulfhydration has also been observed in immune contexts. For example, in regulatory T lymphocytes, TET2 catalytic activity can be indirectly modulated *via* sulfhydration of upstream transcription factors, influencing the course of autoimmune diseases^10^. Such findings highlight the potential of sulfhydration to modulate TET2-dependent immune pathways in cancer. Our previous study revealed that sodium thiosulfate (STS), another representative of active sulfur, has antimelanoma effects^20^. STS is used for various clinical applications, including relieving itching in atopic dermatitis, detoxification after platinum-based chemotherapy, and treating calciphylaxis in chronic kidney disease^21^, ^22^, ^23^. Given TET2’s dual role in epigenetic regulation and tumor immunity, and the ability of active sulfur species to modulate protein function *via* sulfhydration, the possibility that STS may restore TET2 activity and enhance antitumor immunity in melanoma warrants detailed investigation.

In this investigation, we uncovered a previously uncharacterized posttranslational modification of TET2, known as TET2 sulfhydration, which is markedly diminished in melanoma. Treatment with active sulfur restored TET2 protein levels and reprogrammed global DNA hydroxymethylation by repairing sulfhydration at the critical residues C1186 and C1202 in melanoma cells. Reactive sulfur species synergize with anti-PD-1 therapy by augmenting CD8^+^ T cell-mediated antitumor immunity, facilitated by increased IFN-*γ*/STAT1 signaling, which promotes the expression of Th1-type chemokines and MHC-I molecules on melanoma cells.

## 2 Materials and methods

### 2.1 Ethical approval of the study protocol

The study protocol received approval from the Human Ethics Committee of Department of Dermatology, The 3rd Affiliated Hospital of Sun Yat-sen University (02-329-02). All study participants provided written informed consent.

### 2.2 Cell culture and treatment

Human epidermal melanocytes (HEMs) and malignant melanoma cell lines (B16-OVA, A375) were cultured in high-glucose DMEM supplemented with 10% FBS. The cells were maintained at 37 °C in a tissue culture incubator with 5% CO_2_. STS was dissolved in DMEM. B16-OVA and A375 cells were exposed to STS or IFN-*γ* for 24 or 48 h.

### 2.3 Quantitative real-time PCR

Total RNA from cells or tissues was extracted using TRIzol (Invitrogen, USA) and reverse-transcribed into cDNA with the PrimeScript™ RT Reagent Kit with gDNA Eraser (Perfect Real Time) (TaKaRa, Japan). Quantitative RT‒PCR was conducted using the CFX384 system (Bio-Rad, USA) following protocols provided with TB Green® Premix Ex Taq™ II (Tli RNaseH Plus) (Takara, Japan). Changes in the mRNA expression levels of target genes were normalized to those of *β*-actin.

### 2.4 Immunoblotting and immunoprecipitation analysis

Immunoblotting and immunoprecipitation were performed as previously described. Briefly, protein extracts prepared in RIPA lysis buffer were separated on 8%‒10% SDS‒PAGE gels and then transferred to PVDF membranes (Millipore, USA). After blocking, the membranes were incubated with the appropriate primary and secondary antibodies. For CHX half-life experiments, all normalized time points were scaled to 1 by dividing them by the normalized control. For immunoprecipitation, 1 mg of each cell lysates was incubated with the indicated antibody and protein A/G magnetic beads (Bimake, USA) at 4 °C overnight. Eluted immunoprecipitates were subjected to SDS‒PAGE and immunoblotting.

### 2.5 Immunofluorescence staining

Tissues were embedded in Tissue-Tek® O.C.T. Compound (Optimum Cutting Temperature, Sakura Finetek, USA) and frozen immediately. After fixation and blocking, 5 μm sections were incubated with a primary antibody against CD8 (Cat. ab217344, Abcam, UK) at 4 °C overnight and then with a fluorescence-conjugated secondary antibody at 37 °C for 1 h. Images were acquired using a laser scanning microscope system (Nikon Eclipse Ni, Japan).

### 2.6 Maleimide assay for sulfhydration detection

The cells were homogenized in lysis buffer and immunoprecipitated with an anti-TET antibody. After the beads were washed with RIPA buffer, they were incubated with Alexa Fluor 680-conjugated C2 maleimide (2 μmol/L) and incubated at 4 °C for 2 h with occasional gentle mixing. The beads were pelleted and washed with the same buffer, followed by treatment with or without 1 mmol/L DTT for 1 h at 4 °C. The beads were subsequently pelleted again, washed, and suspended in 2 × sodium dodecyl sulfate‒polyacrylamide gel electrophoresis (SDS‒PAGE) buffer for SDS‒PAGE. The proteins were then transferred to PVDF membranes, which were subsequently scanned using the Li-COR Odyssey system. The intensity of the red fluorescence of the TETs was quantified using software integrated into the imaging system.

### 2.7 Mass spectrometry assay

In brief, the immunocomplexes were incubated with protein A/G magnetic beads in binding buffer on a rocking platform, followed by five washes in binding solution. After reduction/alkylation (10 mmol/L DTT at 56 °C for 40 min and 50 mmol/L iodoacetamide for 30 min in the dark), trypsin was added to the gel, and the proteins were incubated overnight at 37 °C. After the indicated treatment with a C18 desalting column, the supernatant was subjected to washing with a solution containing 0.1% formic acid and 4% acetonitrile, followed by elution with a buffer containing 0.1% formic acid and 75% acetonitrile. The eluents were combined and lyophilized and then subjected to LC–MS/MS sequencing and data analysis.

### 2.8 Dot blot

Genomic DNA was isolated using the QIAamp DNA Mini Kit (Qiagen, USA), quantified *via* a NanoDrop 2000 spectrophotometer, and then diluted to the same concentration with ddH_2_O. The extracted DNA was denatured at 95 °C for 10 min and carefully dropped onto NC membranes. After drying at 80 °C for 15 min, each side of the membrane was crosslinked with UV irradiation for 15 min. The blots were blocked with 5% BSA at room temperature for 1 h, followed by overnight incubation with anti-5-mC (Cat. 39649, Active Motif, USA) or anti-5-hmC (Cat. 39069, Active Motif, USA) at 4 °C. The blots were subsequently incubated with the secondary antibody for 2 h. DNA was detected using a Bio-Rad gel imaging system (Bio-Rad, USA), and the dot blot intensity was quantified *via* a gel imaging system.

### 2.9 Reduced representation bisulfite sequencing and oxidative reduced representation bisulfite sequencing

Genomic DNA was extracted using the QIAamp DNA Mini Kit (Qiagen, USA). DNA integrity and concentration were assessed using 1% agarose gel electrophoresis and quantified using the Qubit dsDNA HS Assay Kit (Invitrogen, USA) with the Qubit fluorometer. Subsequently, 2 μg of genomic DNA mixed with unmethylated lambda DNA underwent digestion with the MspI enzyme for 16 h at 37 °C, followed by library construction.

In brief, the purified digested DNA was processed with a mixture of T4 DNA polymerase, Klenow Fragment, and T4 polynucleotide kinase to repair, blunt, and phosphorylate the ends. Next, spike-in controls with 5 mC, 5hmC and 5 fC modification were combined with the blunted DNA. The resulting DNA fragments were then 3′-adenylated using Klenow Fragment (3′–5′ exo-) and ligated to adaptors using T4 DNA Ligase. Before the oxidation reaction, all products were purified using the TrueMethyl Seq Kit (Cambridge Epigenetix, Cambridge, UK), following the manufacturer’s protocols. After purification, the oxidation reaction was carried out using the oxidant solution to create oxidative reduced representation bisulfite sequencing (oxRRBS) libraries. Meanwhile, reduced representation bisulfite sequencing (RRBS) libraries were also prepared as controls, with ultrapure water replacing the oxidant solution. After bisulfite conversion, the final oxRRBS and RRBS libraries were generated through PCR amplification, quantified using an Agilent 2100 Bioanalyzer (Agilent Technologies, USA), and real-time PCR assays before being sequenced in E-GENE Co., Ltd. with the Illumina Novaseq 6000 platform.

### 2.10 Sulfane sulfur detection

Sulfane sulfurs were detected *via* the fluorescent Sulfane Sulfur Probe 4 (SSP4; Cat. SB10, Dojindo Laboratories, Japan). The cells were incubated with the SSP4 probe in serum-free DMEM for 15 min at 37 °C in an incubator. Images were subsequently captured and analyzed using a fluorescence microscope.

### 2.11 Chromatin immunoprecipitation

Chromatin immunoprecipitation was carried out with a ChIP kit (CST, USA) following the manufacturer’s recommended instructions. The genomic DNA and proteins were cross-linked by adding formaldehyde to a final concentration of 1% for 10 min at room temperature. After glycine treatment, the cell chromatin was sonicated to generate fragments ranging from 150 to 900 bp in length. These fragments were then immunoprecipitated using antibodies targeting 5-hmC (Cat. 39069, Active Motif, USA), TET2 (Cat. 92529S, CST, USA), or an isotype control normal rabbit immunoglobulin G (Cat. 2729P, CST, USA). The ChIP-enriched DNA was subsequently analyzed by qPCR using SYBR Green Master Mix.

### 2.12 RNA-sequencing analysis

Total RNA was extracted *via* the TRIzol reagent following the manufacturer’s instructions. The RNA quality was evaluated using the Agilent Bioanalyzer 2100 system (Agilent Technologies, USA). After quality assessment, the mRNA molecules were purified, fragmented, and converted into first-strand and second-strand cDNA. To selectively retain cDNA fragments within the 370 to 420 bp range, library fragments were purified using the AMPure XP system (Beckman Coulter, USA). Following library construction, PCR amplification was subsequently performed. The resulting products were purified and validated using the AMPure XP and Agilent Bioanalyzer 2100 systems, respectively. A cluster of index-coded samples was generated using the TruSeq PE cluster kit v3-cBot-HS (Illumina, USA) on a cBot cluster generation system according to the manufacturer’s instructions.

Raw reads in the fastq format underwent preprocessing to obtain clean reads, which were then aligned to the reference genome sequence of *Mus musculus* using HISAT2 v2.0.5. Gene read counts were calculated using FeatureCounts v1.5.0-p3. Differentially expressed genes (DEGs) between each group were identified using the DESeq2 R package v1.20.0, with significance thresholds set at |log2-fold change (FC)| ≥ 1 and an adjusted *P* value (*P*_adj_) ≤ 0.05. For functional analysis, the ClusterProfiler R software package was utilized to perform Gene Ontology (GO) enrichment analysis and Kyoto Encyclopedia of Genes and Genomes (KEGG) pathway enrichment analysis.

### 2.13 *In vivo* tumor modeling and treatments

All animal studies were conducted with the approval of the Welfare and Ethical Committee for Experimental Animal Care at Southern Medical University. C57BL/6 mice, aged 6 to 8 weeks, were housed under specific pathogen-free (SPF) conditions. B16-OVA cells (2 × 10^5^) were subcutaneously transplanted into the right flank of the mice. Tumor size was assessed using calipers every 2 to 3 days, and tumor volume was calculated using Eq. (1):

(1) Tumor volume = Width^2^× Length × 0.523

The mice were humanely sacrificed when the tumors reached the maximum allowable size (20 mm in diameter).

For the anti-PD-1 immunotherapy model, mice received intraperitoneal injections of 200 μg of anti-PD-1 (Junshi Biosciences) three times per week for two weeks after tumor implantation. In the STS combination treatment group, the mice were intraperitoneally injected with sodium thiosulfate (2 g/kg) or PBS on the indicated days. The tumors were subsequently dissected, photographed, and stored at −80 °C.

### 2.14 Flow cytometry

Single-cell suspensions from the lymph nodes, spleens, blood, and tumors were prepared at the end of the experiment. For T-cell analysis, the cells were treated with a cell stimulation cocktail (including protein transport inhibitors) (Thermo Fisher Scientific, USA) for 5 h before harvest. Following stimulation, the cells were collected and preincubated with Fc receptor blocking solution (Cat. 14-9162-42, Thermo Fisher Scientific, USA) for 10 min at room temperature. The cells were then stained with PerCP/Cyanine5.5 anti-CD3 (Cat. 100217, BioLegend, USA) and Alexa Fluor 594 anti-CD8 (Cat. 100758, BioLegend, USA) antibodies at 4 °C in the dark for 30 min. The cells were subsequently fixed and permeabilized using an intracellular fixation and permeabilization buffer set (Thermo Fisher Scientific, USA) and incubated with Brilliant Violet 510 anti-IFN-*γ* (Cat. 505841, BioLegend, USA) or Brilliant Violet 421 anti-TNF-*α* (Cat. 506327, BioLegend, USA) antibodies at 4 °C for 30 min. To identify live cells, a LIVE/DEAD fixable violet dead cell stain kit (Cat. L34964, Thermo Fisher Scientific, USA) was used. Flow cytometry was conducted using a FACSCelesta flow cytometer (BD Biosciences, Germany), and the data were analyzed using FlowJo software.

### 2.15 Measurement of zinc content by PAR chelation assay

Free zinc was quantified using a 4-(2-pyridylazo)-resorcinol (PAR)-based chelation methodology. Experimental protocols involved treating 40 μg protein samples with 50 μmol/L PAR reagent in cuvettes. Spectral analysis was conducted at 25 °C using a Varian Cary 50 spectrophotometer by scanning 350–550 nm wavelengths. The resorcinol indicator absorbance shifts from 411 to 492 nm in the presence of zinc and the 492 nm peak is recorded and compared to a standard curve for zinc content. The relative zinc content was normalized to protein concentration and expressed by the ratio to the zinc content of the control.

### 2.16 Data source

RNA-seq data were downloaded from the TCGA database (https://tcga-data.nci.nih.gov/tcga/), which contains 459 skin cutaneous melanoma patients. Kaplan-Meier survival curves were used to compare survival rates between different groups.

### 2.17 Statistical analysis

Each experiment was independently replicated a minimum of three times, and the results are presented as the mean ± standard deviation (SD). Comparisons among multiple groups were conducted using one-way analysis of variance (ANOVA). Differences between two groups within individual experiments were assessed using Student’s *t* test. To evaluate correlations between the indicators, Spearman’s correlation analysis was employed. A significance level of *P*< 0.05 was considered statistically significant.

## 3 Results

### 3.1 Loss of sulfhydration of the TET2 protein in melanoma is correlated with clinical stage

**Figure 1:**
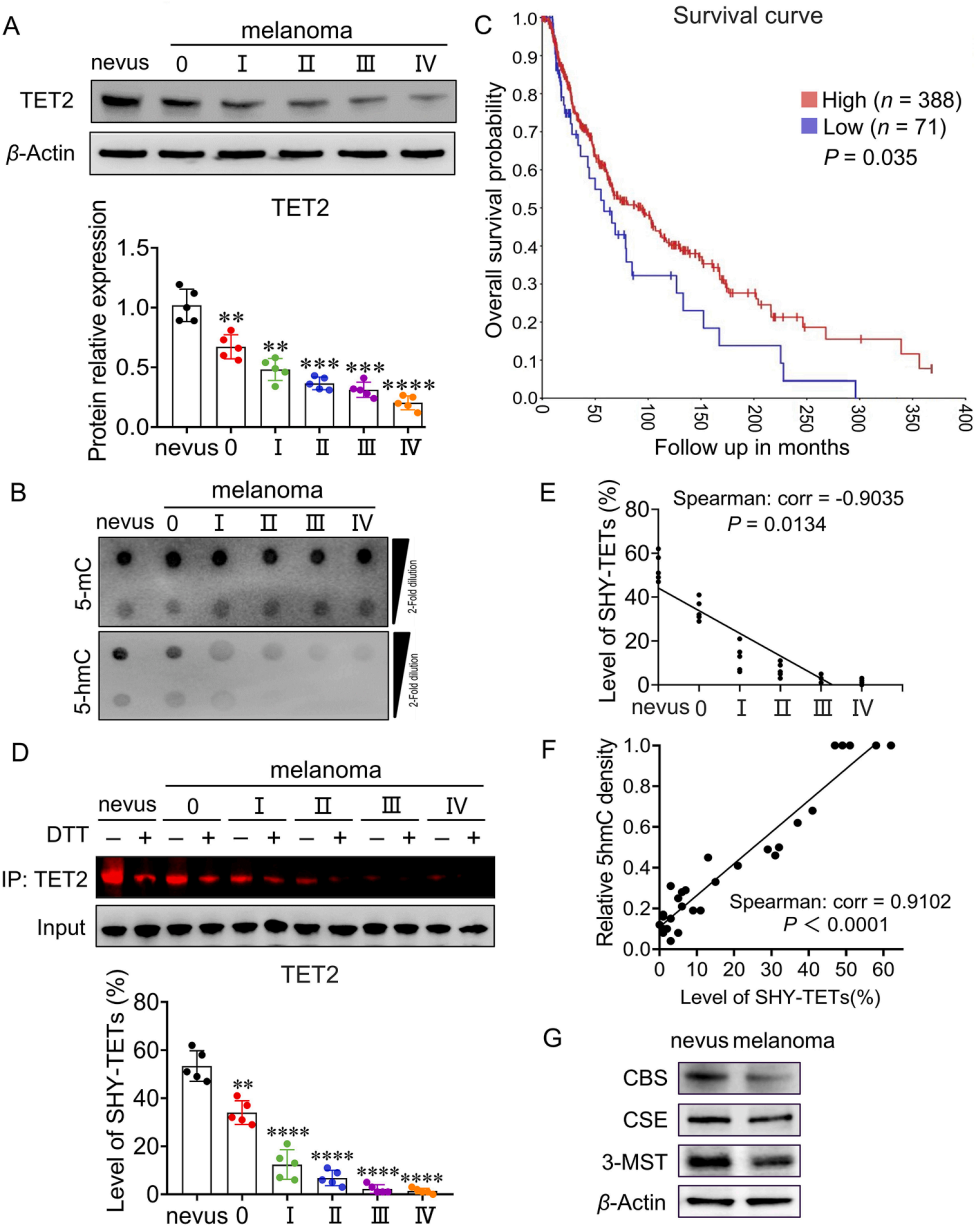
Loss of sulfhydration of TET2 is correlated with the clinical stage of melanoma. (A) TET2 protein expression levels in patients with nevus (*n*= 5) or melanoma (*n*= 25) at different stages. (B) Overall 5-mC and 5-hmC levels in nevus (*n*= 5) and melanoma (*n*= 25) patients. (C) The OS analysis between clinical melanoma patients stratified by low versus high TET2 expression levels (*P*= 0.035, *n*= 459). (D) Detection of the changes in TET2 sulfhydration level along with clinical stage of melanoma. (E) Correlation between TET2 sulfhydration levels and tumor stage. (F) Correlation between TET2 sulfhydration levels and TET2 enzymatic activity. (G) The protein expression levels of CBS, CSE, and 3-MST in nevi and melanoma tissues. All the data are presented as the mean ± SD. Statistical analyses were performed using one-way ANOVA followed by Tukey’s multiple comparisons test for Panels A and D and Spearman’s correlation analysis for Panels E and F. Experiments were conducted in triplicate. ∗∗*P*< 0.01, ∗∗∗*P*< 0.001, ∗∗∗∗*P*< 0.0001.

Decreased TET protein expression and impaired catalytic activity play key roles in the epigenetic pathogenesis of melanoma^4^, prompting us to first measure the levels of mRNA and protein of TET in patients. The results revealed a significant reduction in mRNA and protein levels of TET in patients’ melanoma tissues, with only TET2 protein showing a significant gradient decrease with pathological stage, indicating a negative correlation between TET2 protein levels and melanoma progression (Fig. 1A, Supporting Information Fig. S1A and S1B). Given TET proteins catalyze the conversion of 5-mC to 5-hmC, we next examined global 5-hmC levels using dot blotting. Our data demonstrated a decline in global 5-hmC level, which was negatively correlated with tumor stage (Fig. 1B and Fig. S1C). These findings align with the progressive decline in TET2 protein expression observed during advancing tumor stages. We next performed Kaplan–Meier survival analysis to compare overall survival (OS) in clinical melanoma patients stratified by TET2 expression levels. The results revealed that patients with low TET2 expression exhibited significantly poorer OS compared to those with high TET2 expression (Fig. 1C). These findings underscore the critical role of TET2 in melanoma progression and highlight its prognostic significance in predicting patient outcomes.

*S*-Sulfhydration at cysteine residues has been reported to drive tumor pathogenesis. The zinc-finger structure of TET2 is of interest in this context because it contains a cysteine-rich region, making it a potential target for this modification. Furthermore, the regulation of TET2 by sulfhydration has already been documented in immune contexts. Therefore, we further detected the sulfhydration level of TET2 in melanoma using red maleimide. Lower TET2 sulfhydration level in melanoma was reflected by a slight reduction of red fluorescent intensity after dithiothreitol (DTT; 1 mmol/L) treatment, which can reduce the sulfhydration SSH (thioether bond) to two separate thiol (–SH) groups^10^^,^^24^ (Fig. 1D). Interestingly, TET1 and TET3 did not exhibit significant stage-related phenotypes (Figs. S1B, S1D, and S1E).

Furthermore, we performed correlational analysis between TET2 sulfhydration levels and clinical tumor staging. Our findings revealed that TET2 protein sulfhydration levels were significantly negatively correlated with advanced tumor stage, and positively correlated with 5-hmC expression (Fig. 1E and F). However, we did not observe any significant stage-related phenotypes associated with TET1 or TET3 protein sulfhydration (Fig. S1F). Additionally, we examined the expression of key enzymes catalyzing sulfhydration reactions, including cystathionine-*β*-synthase (CBS), cystathionine-*γ*-lyase (CSE), and 3-mercaptopyruvate sulfurtransferase (3-MST)^10^. The results demonstrated a marked down-regulation of CBS, CSE, and 3-MST in melanoma tissues compared to nevi, suggesting a diminished sulfhydration capacity during melanoma progression (Fig. 1G). Consequently, our study primarily focused on investigating the role of TET2 sulfhydration in this context. These findings illustrate that during melanoma progression there is a gradual decrease in both the sulfhydration and protein levels of TET2.

### 3.2 TET2 sulfhydration enhances its protein stabilization and activity

**Figure 2:**
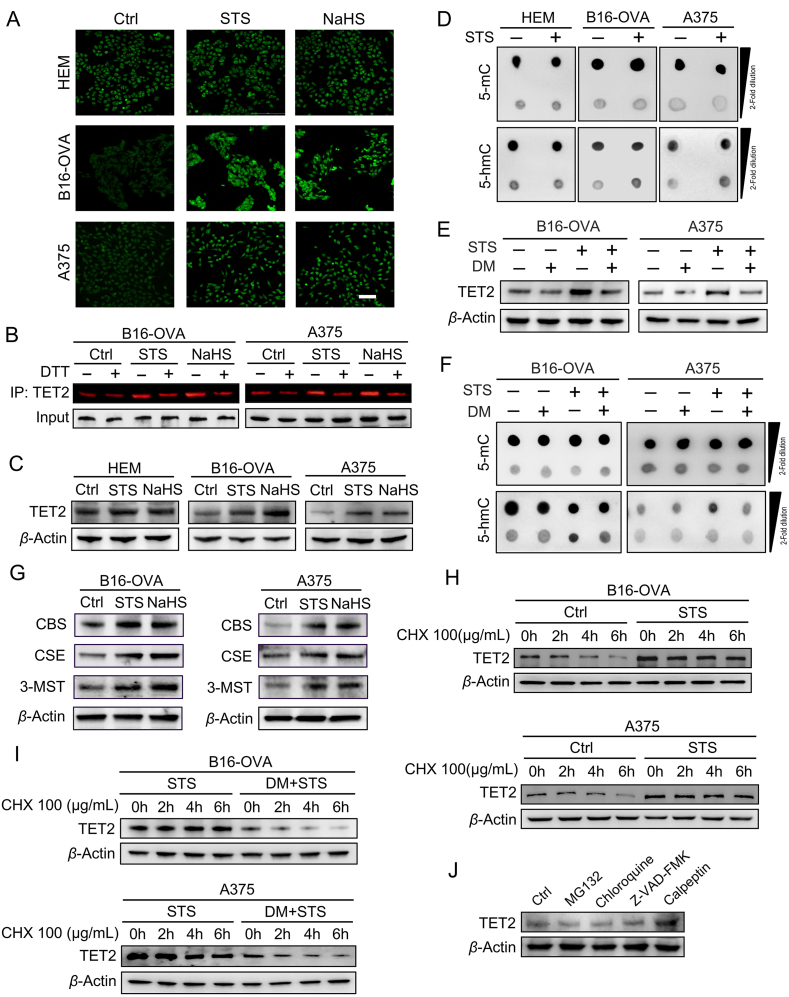
TET2 sulfhydration enhances its protein stabilization and activity. (A) Sulfane sulfur expression levels in normal and malignant cells after active sulfur treatment. The sulfhydrylation level (B) and expression level of TET2 (C) in normal and malignant cells treated with active sulfur for 48 h. (D) Epigenomic profiling of 5-mC and 5-hmC in normal and malignant cells. (E) Comparison of TET2 protein expression levels after stimulation of melanoma cells with the reactive sulfur and sulfhydration inhibitor DM for 48 h. (F) Epigenomic profiling of 5-mC and 5-hmC after the stimulation of melanoma cell lines with reactive sulfur and sulfhydration inhibitor DM. (G) Protein expression levels of CBS, CSE, and 3-MST in different cell types. (H, I) TET2 protein expression in melanoma cell lines following CHX stimulation combined with STS (H) or STS plus DM (I). (J) Treatment effects of MG132, chloroquine, Z-VAD-FMK, and calpeptin on TET2 protein expression.

Next, we utilized human epidermal melanocytes (HEMs) and malignant melanoma cell lines (B16-OVA, A375), which are widely employed in melanoma cell model studies, to investigate alterations in TET2 protein sulfhydration. Sulfane sulfur, a sulfhydration substrate that can be quantified by the SSP4 probe^25^, ^26^, ^27^, ^28^, ^29^, was reduced in melanoma cells compared with the HEM control but restored with active sulfur (1 mmol/L of STS and 100 μmol/L of NaHS) treatment (Fig. 2A). Active sulfur treatment altered both the sulfhydration status and protein levels of TET2 in melanoma cells, without affecting its mRNA expression (Fig. 2B and C, Supporting Information Fig. S2A), suggesting that a decrease in sulfhydration substrate levels led to the loss of sulfhydration and protein expression of TET2 in melanoma cells. Consistent with the results in patients, TET3 protein exhibited lower levels of sulfhydration, and NaHS and STS treatment did not reverse sulfhydration or increase protein levels of TET3 (Fig. S2B–S2D). In contrast, the sulfhydration levels of the TET1 protein could be reversed by NaHS and STS, but its protein level was not fully restored (Fig. S2B–S2D).

After STS treatment, the markedly increased sulfhydration level of TET2 protein aligned with the change in global 5-hmC level of melanoma cells but not HEM (Fig. 2D and Fig. S2E). To further determine the impact of sulfhydration modification on TET2, we treated melanoma cell lines with the sulfhydration inhibitor diamide (DM; 200 μmol/L)^30^. Treatment with DM alone did not alter TET2 expression or catalytic activity, likely due to the intrinsically low basal level of TET2 sulfhydration in melanoma cells. However, when combined with STS, DM effectively inhibited STS-induced TET2 sulfhydration, resulting in a marked reduction in both TET2 protein levels and its catalytic activity (Fig. 2E and F, Fig. S2F–S2G). We also examined the expression of CBS, CSE, and 3-MST, and found that their expression levels were up-regulated following treatment with STS and NaHS (Fig. 2G). These data demonstrate that STS directly reestablishes TET2 sulfhydration in melanoma cells, rescuing both protein expression and catalytic activity.

We then further investigated whether the up-regulation of TET2 protein expression by STS *via* increasing its sulfhydration was attributable to enhanced protein stability. We treated melanoma cell lines with cycloheximide (CHX), which can interfere with protein synthesis^31^, and observed that STS treatment significantly reduced TET protein degradation rates, whereas DM administration enhanced proteolysis (Fig. 2H and I, Fig. S2H and S2I). Our findings demonstrate that STS-induced sulfhydration stabilizes TET2 in melanoma cells. We subsequently treated cells with inhibitors of the four major proteolytic pathways, including MG132, a proteasome inhibitor; chloroquine, a lysosomal inhibitor; Z-VAD-FMK, a caspase inhibitor; and the calpain-specific inhibitor calpeptin, to investigate which pathway mediates TET2 degradation. The results suggested that the calpain system is a major contributor to TET2 proteolysis (Fig. 2J).

Given the role of active sulfur in regulating TET2 activity *via* sulfhydration, we propose that active sulfur, such as STS, could offer therapeutic benefits in melanoma through this process. To test this hypothesis, we constructed a melanoma cell line with *Tet2* knockout using CRISPR-Cas9 technology (Supporting Information Fig. S3A). Consistent with previous research^2^, no proliferative changes were observed in *Tet2*-deficient melanoma cells *in vitro* (Fig. S3B). However, *in vivo* treatment of wild-type *Tet2* melanoma-bearing mice with STS (2 g/kg) significantly inhibited tumor growth, whereas this inhibition was lost after *Tet2* knockout (Fig. S3C–S3F), indicating that STS exerts this inhibition through TET2 in mice. Additionally, STS did not induce severe toxicity in the mice throughout the treatment process (Fig. S3G and S3H). To determine whether STS affects tumor growth in mice by influencing TET2 sulfhydration, we used DM (200 mg/kg) to inhibit sulfhydration. The results showed that DM could block the therapeutic effects of STS (Fig. S3I–S3M). Moreover, *in vivo*, STS increased TET2 protein levels and catalytic activity, whereas DM blocked these effects (Fig. S3N–S3P). Overall, these results highlight the potential of STS as a therapeutic agent to inhibit melanoma growth through the modulation of TET2 sulfhydration.

### 3.3 Active sulfur sulfhydrates the C1186 and C1202 dual sites to stabilize TET2 protein expression

**Figure 3:**
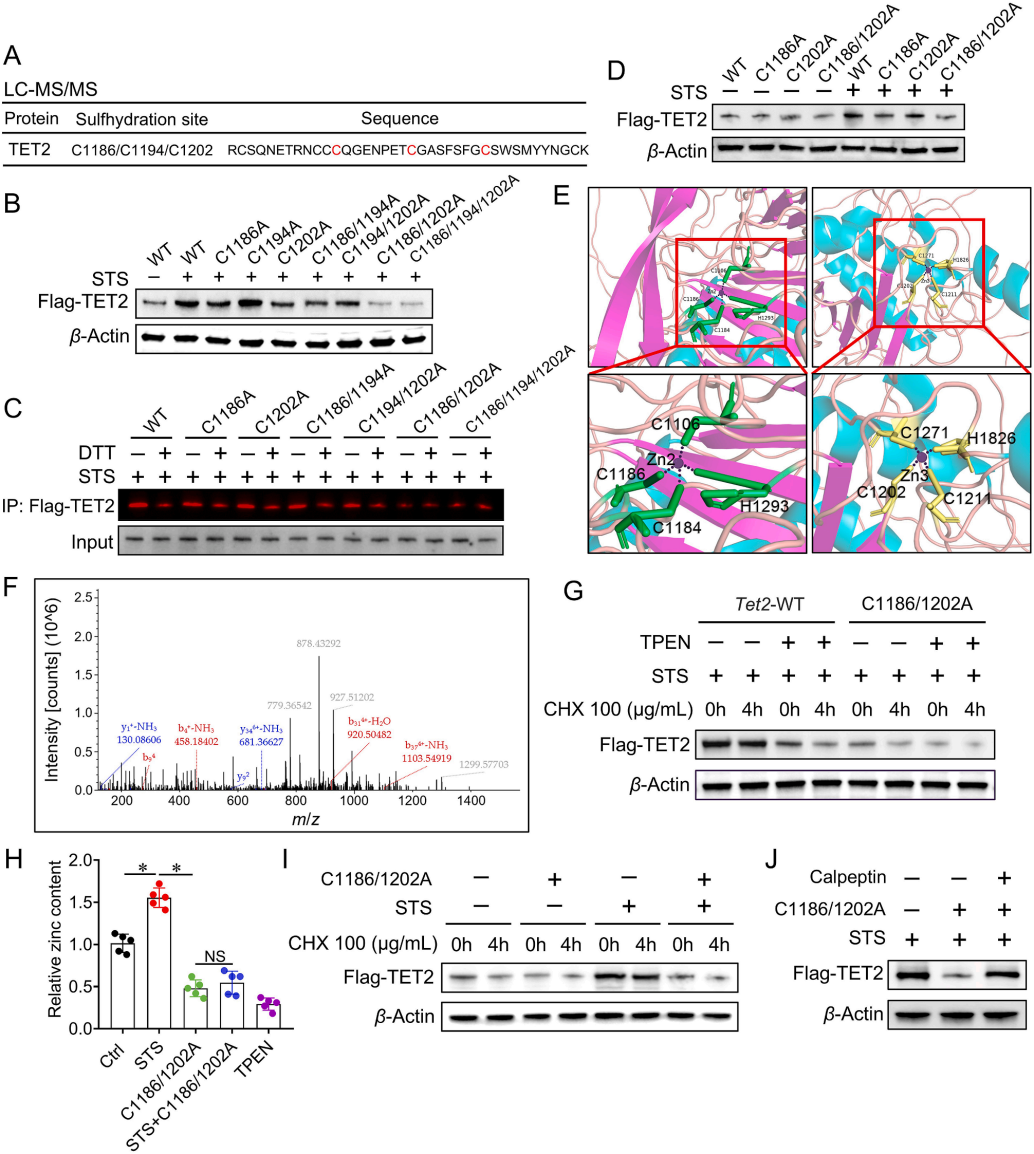
Active sulfur sulfhydrates the C1186 and C1202 dual sites to stabilize TET2 protein expression. (A) LC‒MS/MS analysis of sulfhydration of the TET2 protein Cys1186, 1164, and 1202 after active sulfur treatment. (B) TET2 protein expression and (C) sulfhydration in melanoma cells after the indicated treatments for 48 h. (D) Quantitative measurement of TET2 protein in genetically distinct melanoma cells with or without STS. (E, F) Schematic diagram of the structure of the TET2 sulfhydration site. (G) TET2 protein expression under STS and CHX stimulation, with or without TPEN treatment in the indicated groups. (H) TPEN-induced reduction of zinc content in TET2 protein, expressed as the ratio of zinc content in the treatment group to that in the control (*n*= 5). (I) TET2 protein expression following site-specific mutation under CHX stimulation with or without STS treatment. (J) TET2 protein expression under STS stimulation, with or without calpeptin treatment, in *Tet2*-WT and mutant groups. Data are presented as mean ± SD from three independent experiments. Statistical comparisons were performed using one-way ANOVA with Tukey’s *post hoc* test. ∗*P*< 0.05, NS indicates not significant.

To identify specific sulfhydration sites, we performed liquid chromatography‒mass spectrometry/mass spectrometry (LC‒MS/MS)^32^, which revealed three candidate cysteines, C1186, C1194, and C1202 (Fig. 3A and F). Site-directed mutagenesis showed that mutation of C1186A or C1202A, particularly in combination, abolished STS-induced TET2 up-regulation, whereas C1194A had no effect (Fig. 3B–D). Consistently, sulfhydration levels were significantly reduced in C1186A and C1202A mutants, but not in C1194A. AlphaFold structural predictions further indicated that C1186 and C1202 were involved in maintaining the TET2 zinc finger structure (Fig. 3E), in agreement with previous studies^33^.

Given that the zinc ion coordination capacity of zinc finger domains critically governs their folding compactness and functional competence^33^, we then explored whether the sulfhydrated cysteine residues within the zinc finger domain profoundly impacted the DNA binding capacity and enzymatic activity of TET2 through regulating zinc coordination. We found that STS enhanced zinc binding in wild-type but not mutant TET2. Consistently, wild-type TET2 exhibited significantly slower degradation rates compared to C1186/C1202A counterparts under STS stimulation combined with zinc ion chelation created by *N*,*N*,*N*′,*N*′-tetrakis (2-pyridylmethyl) ethylenediamine (TPEN; 5 μmol/L)^34^, a membrane-permeable zinc chelator (Fig. 3G and H). These findings suggest that sulfhydration at C1186 and C1202 stabilizes TET2 protein by enhancing zinc-binding capacity and thereby preserving zinc finger spatial configuration.

To determine whether C1186/C1202 sulfhydration of TET2 protein protects against calpain-mediated degradation, we engineered melanoma cells expressing the TET2 C1186/C1202A mutant. In these cells, STS treatment failed to stabilize TET2, while calpain inhibition restored TET2 levels to those observed in STS-treated wild-type cells, indicating that C1186/C1202 sulfhydration effectively limits calpain-dependent TET2 degradation (Fig. 3I and J). These findings provide crucial mechanistic insights into calpain-mediated TET2 proteolysis, suggesting that insufficient sulfhydration modifications in melanoma cells may lead to improper zinc finger folding. This structural destabilization may expose calpain’s catalytic cleavage sites within the core domain, rendering TET2 susceptible to proteolysis. While this hypothesis aligns with our experimental observations, it requires more direct evidence regarding spatial conformation changes in the zinc finger domain of TET2 protein.

### 3.4 Active sulfur has a synergistic effect with anti-PD-1 therapy *in vivo*

**Figure 4:**
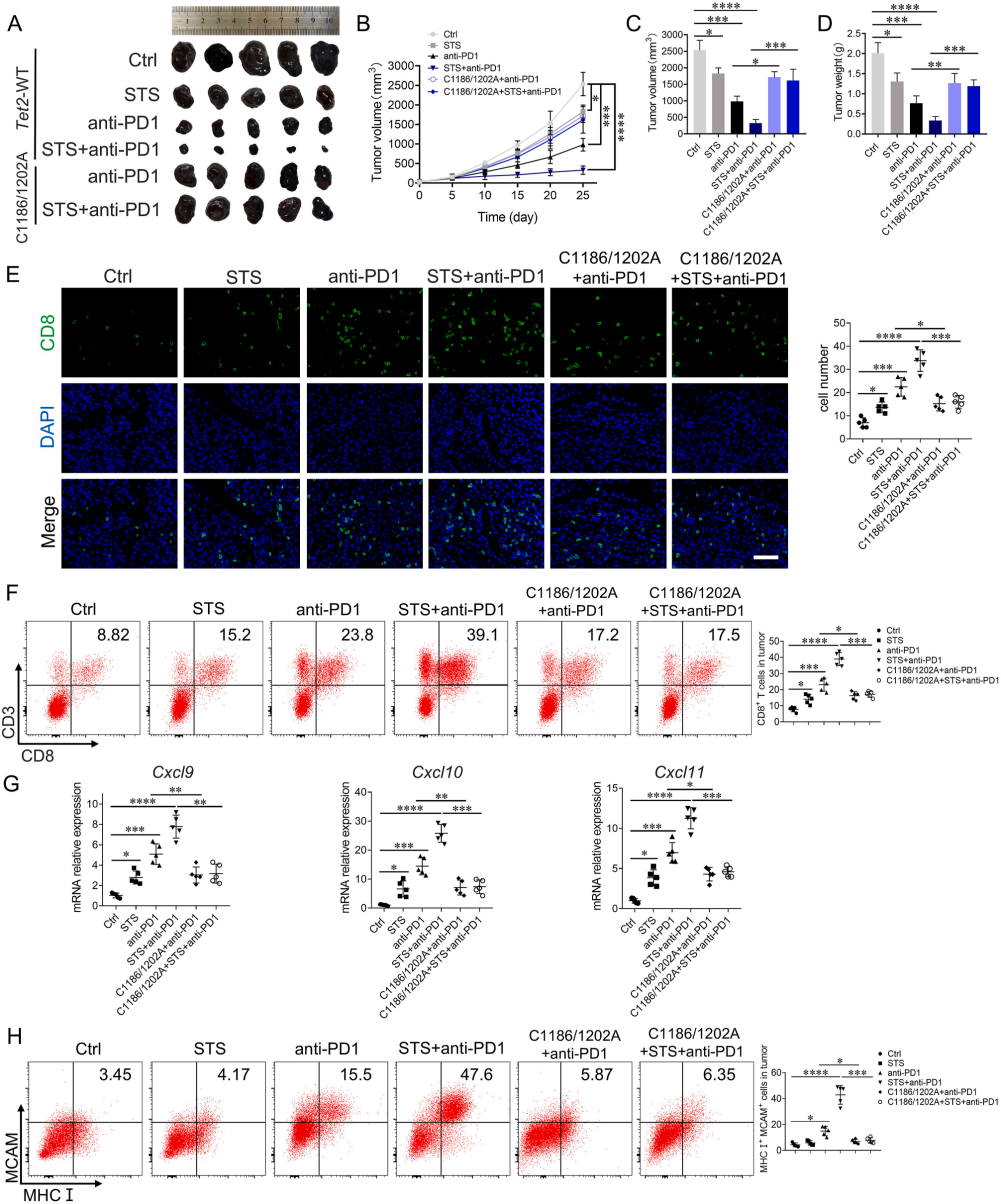
Active sulfur has a synergistic effect with anti-PD-1 therapy *in vivo.* (A) Tumor size, (B, C) tumor volume, and (D) tumor weight of melanoma tumor-bearing mice in the indicated groups. IF (E) and flow cytometry (F) were used to detect the proportion of CD8^+^ T lymphocytes in the tumor tissues of the mice in the indicated groups. (G) mRNA levels of *Cxcl9*, *Cxcl10*, and *Cxcl11* in tumor-bearing mouse tissues from the indicated groups. (H) Proportion of MHC-Ⅰ^+^MCAM^+^ melanoma cells in the mice in the designated groups. For the anti-PD-1 immunotherapy model, mice received intraperitoneal injections of 200 μg of anti-PD-1 three times per week for two weeks after tumor implantation. In the STS combination treatment group, the mice were intraperitoneally injected with STS or PBS on the indicated days. Values are expressed as mean ± SD (*n*= 5 mice/group) from triplicate experiments, with intergroup differences evaluated by one-way ANOVA incorporating Tukey’s multiple comparison adjustment. ∗*P*< 0.05, ∗∗*P*< 0.01, ∗∗∗*P*< 0.001, ∗∗∗∗*P*< 0.0001.

Recent studies have identified TET enzymatic activity as both a predictive biomarker for PD-1 blockade response and a potential immunomodulatory target^2^. We found that STS induces TET2 sulfhydration, restoring its stability and activity in melanoma cells. Accordingly, STS significantly enhanced the antitumor efficacy of anti-PD-1 therapy in *Tet*2-WT tumor-bearing mice but not in those with the TET2 C1186/C1202A mutation (Fig. 4A–D). The combination therapy yielded a coefficient of drug interaction (CDI) of 0.58, indicating a strong synergy dependent on TET2 sulfhydration (Supporting Information Fig. S4A).

PD-1 blockade enhances antitumor immunity by promoting CD8^+^ T cell infiltration^35^. Combination therapy further increased CD8^+^ T cell accumulation in tumors, blood, and lymph nodes, an effect markedly impaired in TET2 mutant models (Fig. 4E and F, Fig. S4B and S4C). Mechanistically, the dual treatment up-regulated Th1-type chemokines (*Cxcl9*, *Cxcl10*, and *Cxcl11*) which facilitate T cell recruitment (Fig. 4G)^36^, and synergistically enhanced tumor MHC-I expression (Fig. 4H), a key determinant of immune checkpoint response^37^. Compared to C1186/C1202A TET2, wild-type TET2 under STS treatment exhibited greater enrichment at the promoter of interferon regulatory factor 1 (IRF1), a key transcription factor directly binding to the MHC-I gene promoter. Consistently, the mRNA expression of IRF1 was markedly increased in the STS-treated melanoma cells (Fig. S4D and S4E), suggesting that STS-driven TET2 sulfhydration epigenetically up-regulates IRF1 transcription, which consequently augments MHC-I presentation in melanoma cells during combined treatment. Collectively, these findings demonstrate STS performs as a TET2-dependent enhancer of anti-PD-1 immunotherapy through epigenetic Th1-type chemokine induction and MHC-I up-regulation, ultimately promoting CD8^+^ T cell infiltration and antitumor immunity.

### 3.5 Active sulfur enhances CD8^+^ T-cell activation and inhibits CD8^+^ T-cell exhaustion to potentiate the antitumor activity of PD-1 blockade

**Figure 5:**
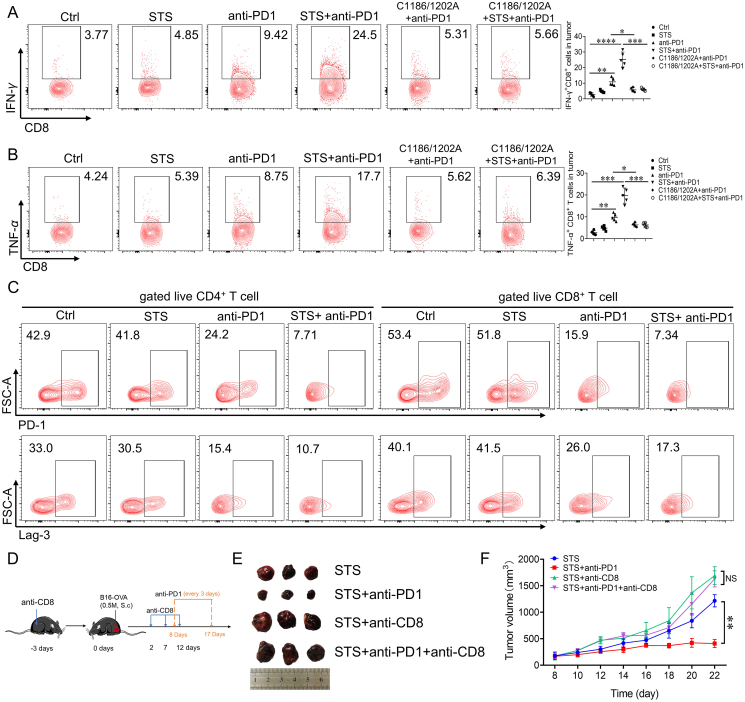
Active sulfur enhances CD8^+^ T-cell activation inhibits CD8^+^ T-cell exhaustion to potentiate the antitumor activity of PD-1 blockade. (A, B) Proportion of IFN-*γ*^+^ CD8^+^ T cells (A) and TNF-*α*^+^ CD8^+^ T cells (B) in melanoma tumor-bearing mouse tissue in the indicated groups. (C) Flow cytometric analysis was performed to evaluate immune checkpoint molecule expression (PD-1^+^Lag-3^+^) on tumor-associated CD4^+^ and CD8^+^ T lymphocytes across experimental cohorts. (D) An illustrative overview of the treatment protocol. (E) Tumor size and (F) tumor volume of melanoma tumor-bearing mice in the indicated groups. The data are presented as the mean ± SD (*n*= 5 mice/group). Statistical analyses were conducted using one-way ANOVA with Tukey’s *post hoc* test for multiple comparisons, with data representing three biological replicates. ∗*P*< 0.05, ∗∗*P*< 0.01, ∗∗∗*P*< 0.001, ∗∗∗∗*P*< 0.0001; NS indicates not significant.

The antitumor efficacy of PD-1 blockade is significantly attributed to the activation of CD8^+^ T cells, which secrete IFN-*γ* and TNF-*α* as critical effector molecules^35^. We discovered that when combined with PD-1 blockade, STS enhances the secretion of IFN-*γ* and TNF-*α* by CD8^+^ T cells in the tumor microenvironment, blood, spleen, and lymph nodes. This effect was significantly blocked in TET2 C1186/C1202A mutant tumor-bearing mice, not only in response to anti-PD-1 single-agent treatment but also in reaction to the combination treatment (Fig. 5A and B, Supporting Information Fig. S5A–S5F). Subsequent analysis of T-cell exhaustion markers revealed that STS and PD-1 blockade combination therapy markedly decreased terminally exhausted CD8^+^ T cell populations (PD-1^+^Lag-3^+^) in *Tet2*-WT tumors, compared to either treatment alone (Fig. 5C). CD8^+^ T cell depletion experiments using neutralizing antibodies completely abrogated the antitumor efficacy of both PD-1 monotherapy and combination treatment in B16-OVA models (Fig. 5D–F), demonstrating CD8^+^ T cell-dependence for therapeutic response. Collectively, these findings demonstrate that active sulfur potentiates PD-1 blockade efficiency by promoting the CD8^+^ T cell effector function and combating the exhaustion pathways.

### 3.6 Active sulfur reprograms IFN-γ-induced global DNA hydroxymethylation and activates key immune signaling pathways in melanoma

In the tumor immune microenvironment, Th1-type chemokines are mainly triggered by IFN-*γ*, and PD-1 blockade enhances IFN-*γ* production in CD8^+^ cytotoxic T lymphocytes^36^. These findings position TET2 as a potential facilitator of anti-PD-1-induced IFN-*γ* production in CD8^+^ T cells, subsequently driving Th1-type chemokine responses. Mechanistically, STS potentiates the immunostimulatory effects of PD-1 blockade, augmenting IFN-*γ* production by CD8^+^ T lymphocytes across multiple compartments including the tumor niche, circulation, and lymphoid organs. Notably, this effect is attenuated in tumor-bearing mice with *Tet2* knockout (Supporting Information Fig. S5A‒S5C). IFN-*γ* has been implicated in inducing epigenetic remodeling in melanoma, whereas TET2 plays a crucial role as a catalytic-dependent mediator of IFN-*γ*-induced immune regulation in melanoma^2^^,^^38^. Having confirmed that the regulation of sulfhydration by STS enhances TET2 expression and activity (Fig. S3), which is crucial for its anti-melanoma effect, and noting that TET2 influences target gene expression mainly through modulating DNA demethylation, we next aim to explore whether STS-mediated TET2 sulfhydration remodels IFN-*γ*-induced epigenetic changes by regulating 5-hmC levels, and to identify the specific downstream target genes involved.

**Figure 6:**
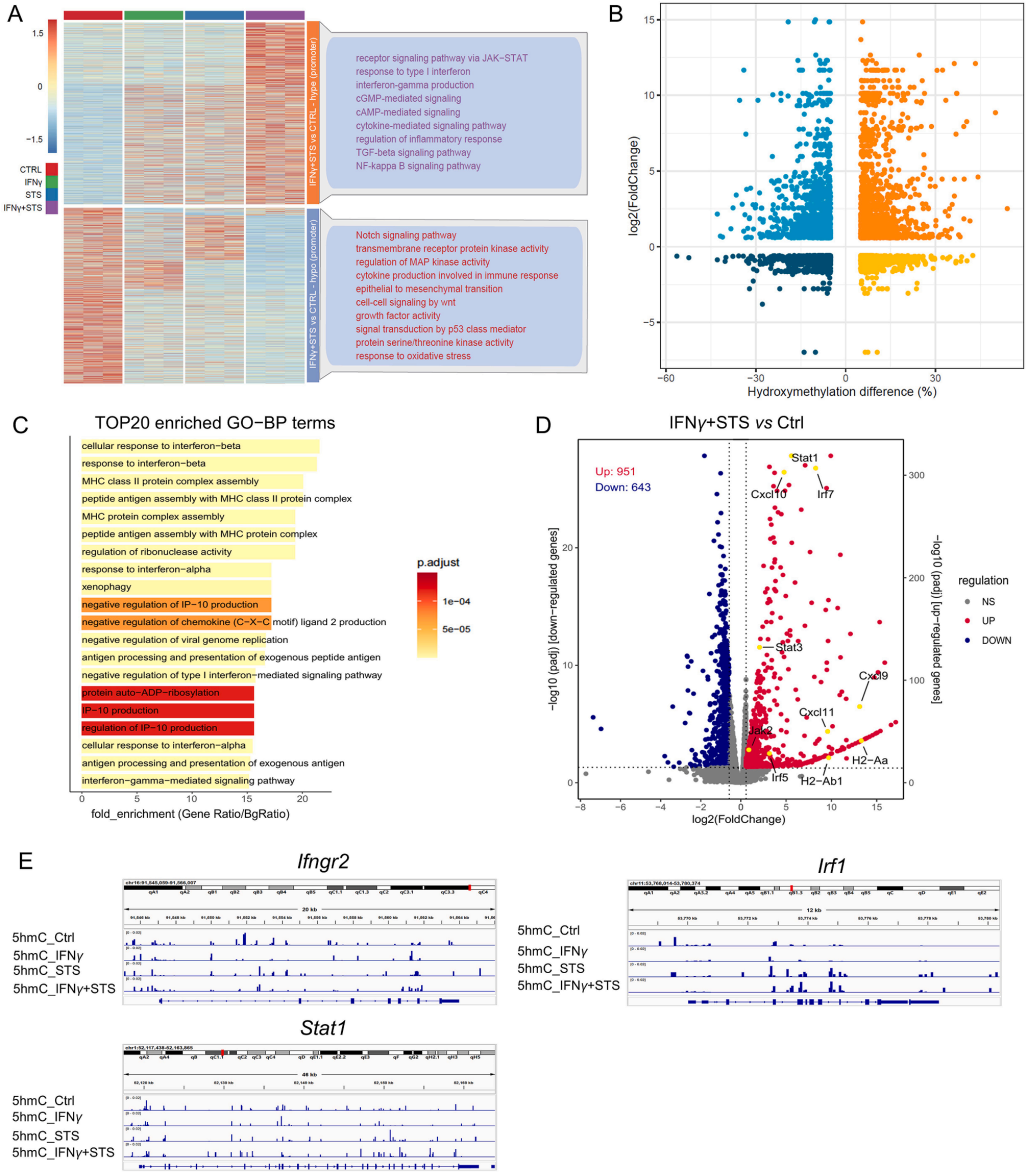
Active sulfur reprograms global DNA 5-hmC levels and activates key immune signaling pathways in melanoma. (A) Heatmap showing DhMRs in melanoma cell lines subjected to the indicated treatments and the results of gene enrichment signaling pathway analysis *via* GO analysis. (B) Analysis of the hydroxymethylation data together with RNA-seq data revealed that the differentially expressed genes (DEGs) were regulated by DNA hydroxymethylation. (C) GO enrichment analysis of the signaling pathways associated with the upregulated genes associated with DNA hydroxymethylation after comparison between the control group and the dual therapy group. (D) Volcano plot of differences in gene expression between the single-agent group and the dual therapy group. (E) The distribution of 5-hmC densities in the indicated genes by RRBS and oxRRBS. Experimental data represent mean ± SD from triplicate biological replicates, with statistical analysis conducted using one-way ANOVA and Tukey’s *post hoc* test.

We conducted a comprehensive analysis of 5-hmC changes in B16-OVA cells *via* reduced representation bisulfite sequencing (RRBS) and oxidative reduced representation bisulfite sequencing (oxRRBS)^39^. Importantly, a significant alteration in the overall hydroxymethylation level was detected in B16-OVA cells in the dual therapy group compared with the monotherapy group, and the 5-hmC level in the dual therapy group was significantly greater than single agent group (Fig. 6A). Subsequently, we further explored the signaling pathway changes related to differentially hydroxymethylated region (DhMR)-enriched genes. Notably, the genes that gained hydroxymethylation were mainly immune-related genes, whereas those that lost hydroxymethylation were predominantly cancer-associated genes (Fig. 6A). Functional annotation of differentially hydroxymethylated genes through gene ontology and disease ontology classification demonstrated that those genes with preferentially distributed hydroxymethylation were enriched mainly in tumor immune pathways regulated by JAK–STAT, type I interferon, and cAMP/cGMP-mediated signaling (Fig. 6A). Conversely, genes exhibiting hydroxymethylation depletion showed significant enrichment in oncogenic pathways, particularly Notch signaling, Wnt cascade, and p53 regulatory networks (Fig. 6A).

Next, we cross-analyzed the hydroxymethylation data and RNA-seq data and revealed that a total of 1594 genes were regulated by DNA hydroxymethylation, including 951 genes whose expression was up-regulated and 643 genes whose expression was down-regulated due to the loss of DNA hydroxymethylation (Fig. 6B). Further functional analysis revealed an increase in antitumor immune-related functions following combined treatment (Fig. 6C). The significantly up-regulated genes included those related to chemokines (*Cxcl9*, *Cxcl10*, *Cxcl11*), transcriptional regulators (*Stat1*), and immune surveillance (*H2-Ab1*, *H2-Aa*, *Jak2*) (Fig. 6D). We further performed 5-hmC enrichment on genes involved in immune- and cancer-related pathways. Comprehensive profiling of 5-hydroxymethylcytosine patterns was performed across JAK–STAT signaling components. We found that the combined treatment notably elevated the 5-hmC levels of genes such as *Ifngr2*, *Stat1*, and *Irf1* (Fig. 6E). Conversely, combined treatment significantly decreased the 5-hmC levels of typical melanoma-associated genes, such as *Nras*, *Mdk*, *Ephb2*, *Pik3r1*, *Akt1* and *Mtor*. (Supporting Information Fig. S6A and S6B). In summary, these results suggest that active sulfur affects the overall DNA hydroxymethylation pattern induced by IFN-*γ* and activates critical immune signaling pathways in melanoma.

### 3.7 TET2 sulfhydration promotes its recruitment by STAT1 to increase IFN-γ-induced Th1-type chemokine production

**Figure 7:**
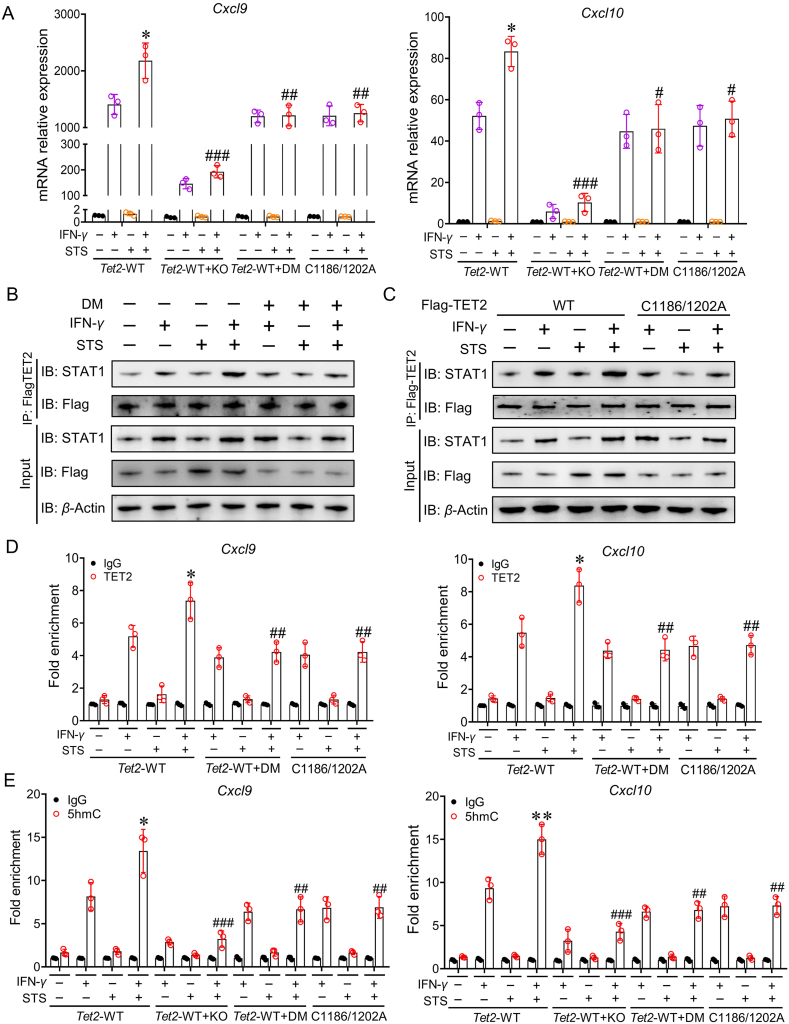
TET2 sulfhydration promotes its recruitment by STAT1 to increase IFN-*γ*-induced Th1-type chemokine production. (A) mRNA expression of *Cxcl9* and *Cxcl10* in WT and C1186/1202 mutant cells following treatment with STS, IFN-*γ*, or combination therapy. (B, C) STS combined with IFN-*γ* enhanced the association between TET2 and STAT1, and DM (B) and the C1186/1202 mutation (C) in TET2 significantly reversed these effects. (D) ChIP‒qPCR was used to detect the binding of the TET2 protein to the promoters of the chemokines *Cxcl9* and *Cxcl10* in WT and C1186/1202 mutant cells following treatment with STS, IFN-*γ*, or combination therapy. (E) ChIP‒qPCR was used to detect 5-hmC levels at the *Cxcl9* and *Cxcl10* promoters in WT and C1186/1202 mutant cells following treatment with STS, IFN-*γ*, or combination therapy. Experimental data represent mean ± SD from triplicate biological replicates, with statistical analysis conducted using one-way ANOVA and Tukey’s *post hoc* test. ∗*P*< 0.05, ∗∗*P*< 0.01.

Previous research highlights TET2 as a crucial mediator in the induction of several Th1-type chemokines in response to IFN-*γ* stimulation, which mediates tumor sensitivity to PD-1/PD-L1 checkpoint blockade^2^^,^^38^. Therefore, we investigated the influence of TET2 sulfhydration on IFN-*γ*-induced Th1 chemokine production. Experimental conditions included IFN-*γ* (10 ng/mL) exposure, STS administration, or co-treatment in B16-OVA tumor cells. Notably, single-agent STS treatment showed minimal impact on *Cxcl9*/*Cxcl10* expression (Fig. 7A). Conversely, IFN-*γ* alone markedly increased the expression of these chemokines (Fig. 7A). STS synergistically amplified IFN-*γ*-stimulated chemokine production in *Tet2*-WT cells, while *Tet2*-deficient systems showed blunted responses to both IFN-*γ* monotherapy and combination treatment (Fig. 7A). These results imply that STS enhances the expression of Th1 chemokines in an IFN-*γ*-dependent manner. Additionally, we observed that treatment with DM or the C1186 and C1202 mutations in TET2 significantly reversed the induction of chemokines by the combined treatment (Fig. 7A). These results imply that STS modulates the sulfhydration of TET2 to increase IFN-*γ*-induced Th1-type chemokine production.

The Th1-type chemokines activated by IFN-*γ* rely primarily on signal transducers of JAKs/STATs^40^. STAT1 can recruit TET2 to promote Th1-type chemokine activation^2^. These findings lead us to speculate that TET2 sulfhydration enhances its recruitment by STAT1 to enhance IFN-*γ*-stimulated Th1-associated chemokine production. This hypothesis was validated by immunoprecipitation assays, which showed low-level TET2:STAT1 complexes in untreated cells, with IFN-*γ*/STS co-treatment potentiating this association (Fig. 7B and C). Additionally, the C1186/1202 mutation of TET2 or treatment with DM significantly reversed the enhanced binding between TET2 and STAT1 induced by the combined treatment (Fig. 7B and C). Furthermore, concurrent STS and IFN-*γ* stimulation markedly enhanced TET2 occupancy at *Cxcl9* and *Cxcl10* promoter regions, as demonstrated by chromatin immunoprecipitation (ChIP‒qPCR) (Fig. 7D). However, the C1186/1202 mutation and DM treatment significantly reversed these effects (Fig. 7D). Similarly, after the combined treatment, the levels of 5-hmC on the *Cxcl9* and *Cxcl10* promoters were significantly increased by 12-fold and 16-fold, respectively, in the *Tet2-*WT cells, whereas this increase was blocked by the C1186/1202 mutation in the *Tet2*-KO cells and in the cells treated with DM (Fig. 7E). These findings underscore the critical importance of sulfhydration at the TET2 site C1186/1202 for promoting TET2 binding with STAT1 to hydroxymethylate Th1-related chemokines.

In summary, our findings delineate a molecular cascade whereby STS-mediated TET2 sulfhydration facilitates STAT1-dependent recruitment, thereby enabling IFN-*γ*-driven Th1 chemokine induction in melanoma cells - a process that ultimately enhances tumor immunogenicity and potentiates PD-1 checkpoint blockade efficacy.

## 4 Discussion

The diminished expression of the DNA demethylase TET2 protein, which leads to aberrant DNA hydroxymethylation, has been identified as a crucial epigenetic event in melanoma pathogenesis^4^^,^^41^. The TET2 protein and its catalytic activity are regulated by a variety of PTMs. As a novel PTM, protein *S*-sulfhydration, can modulate protein activity and function, playing a pivotal role in intracellular signaling pathways^42^^,^^43^. Recent studies have indicated that various active compounds, including glutamine, taurine, and methionine, are related to tumor immunity^44^, ^45^, ^46^. Our investigation reveals a pivotal link between sulfane sulfide levels and TET2 sulfhydration, shedding light on a crucial regulatory mechanism in melanoma. We observed that reduced sulfane sulfide levels in melanoma are associated with a marked decrease in TET2 sulfhydration, leading to aberrant DNA hydroxymethylation patterns. Notably, the reintroduction of active sulfur was able to restore TET2 sulfhydration in melanoma cells specifically through sulfhydration at the critical dual sites of C1186/1202. Sulfhydration at the C1186/1202 sites is indispensable for maintaining the functional integrity of TET2. This sulfhydration not only restores the stability and catalytic activity of TET2 but also normalizes global DNA hydroxymethylation in melanoma cells.

Importantly, we provide crucial insights into the mechanism by which sulfhydration specifically regulates TET2 protein stability. Given that the primary sulfhydration sites (C1186/C1202) are located within the zinc finger domain of TET2, and that zinc coordination is critical for maintaining proper spatial folding, we found that STS stimulation significantly increased zinc-binding capacity in wild-type TET2, but not in the sulfhydration-deficient mutant. Correspondingly, wild-type TET2 exhibited slower degradation under zinc chelation, whereas the mutant form remained unstable. These findings indicate that sulfhydrated cysteine residues strengthen the compactness of zinc finger folding core and thereby stabilize TET2 protein. Insufficient sulfhydration in melanoma may thus result in structural destabilization and accelerate the degradation of TET2.

Compared to TET2, TET1 contains an N-terminal CXXC zinc-finger domain that is absent from TET2. And structural and sequence analyses indicate that the cysteine distribution within the cysteine-rich domain of TET2 is not fully conserved in TET1^33^. These structural differences may therefore help to explain why TET2, but not TET1, is susceptible to sulfhydration-mediated stabilization. It is important to note, however, that there is currently no direct experimental evidence comparing cysteine accessibility and sulfhydration potential across the TET family. Future studies will be required to definitively establish if these domain-specific cysteine features underlie the unique sulfhydration-mediated stabilization observed in TET2.

Our findings are important for understanding how the PTM of TET2 contributes to the pathogenesis of melanoma, potentially introducing an immunotherapeutic strategy to address resistance to immunotherapy^47^, ^48^, ^49^. The correlations observed between TET sulfhydration levels and melanoma tumor stage, TET activity, and response to immunotherapy underscore the potential for a combined therapeutic approach. Previous study establishes TET enzyme function as both a predictive indicator for immunotherapy responsiveness and a modifiable determinant of antitumor immunity, with ascorbate-mediated TET2 activation shown to potentiate PD-L1 blockade efficacy in murine cancer models^2^. Our preclinical animal investigations demonstrated a significant synergistic impact of STS treatment alongside PD-1 immunotherapy. STS-induced TET2 sulfhydration not only restores the stability and catalytic activity of TET2 in melanoma cells but also potentiates IFN-*γ*-related antitumor immunity, including Th1-type chemokine production and MHC-I expression, in melanoma cells, thereby increasing tumor-related CD8^+^ T-cell activation and inhibiting its exhaustion to increase PD-1 immunotherapeutic efficacy. Furthermore, STS has widely been used for various clinical applications, including detoxification after platinum-based chemotherapy and the treatment of calciphylaxis in chronic kidney disease^21^, ^22^, ^23^. These preclinical findings position STS as a promising immunoadjuvant candidate for combinatorial melanoma therapy, warranting clinical evaluation to enhance PD-1 checkpoint blockade efficacy.

This work establishes TET enzymatic deficiency as a novel immune evasion mechanism in melanoma, conferring resistance to PD-1 checkpoint blockade through impaired antitumor immunity. Knocking out *Tet2* nullifies the antitumor effect of the combined treatment, underscoring TET2’s pivotal role in the therapeutic approach involving active sulfur combined with immunotherapy for melanoma. Conversely, the restoration of TET2 sulfhydration restores tumor immune surveillance and enhances immunotherapeutic efficacy, which is attributed to TET2 sulfhydration promoting its recruitment by STAT1 to enhance IFN-*γ*-related antitumor immunity, including Th1-type chemokine production and MHC-I expression, in melanoma cells.

Interestingly, we demonstrated STS-mediated tumor growth suppression through Wnt/*β*-catenin signaling in nude mice in our another pre-clinical study^20^. Considering that our current work demonstrates STS-regulated TET2 mainly executes its anti-melanoma effects through two mechanisms: facilitating tumor infiltration by T-lymphocytes and augmenting their cytotoxic capabilities, and given that nude mice possess inherent deficiencies in T-cell mediated immunity, we postulate that STS predominantly employs TET2-dependent pathways to mediate antitumor responses in immunocompetent systems. The Wnt/*β*-catenin pathway may represent an alternative mechanism operative specifically in immunodeficient contexts.

## 5 Conclusions

In conclusion, our study not only elucidates that TET2 sulfhydration serves as a critical modification for protein stability and plays an essential role in maintaining epigenetic and immune integrity in melanoma but also advocates for the combined treatment of active sulfur such as STS and anti-PD-1 mAbs in melanoma, presenting innovative concepts and strategies for melanoma immunotherapy.

## Author contributions

Yunsheng Liang and Di Wang conceived and designed the research. Di Wang, Yang Xie, Ansheng Xie, Yuan Chang, Shuheng Li, Yishan Chen, and Leiqiong Gao performed the experiments. Yunsheng Liang, Weiwei Deng, Di Wang, Lian Zhang, Yuan Chang, Yan Zhang, and Bocheng Wang analyzed the data. Yang Xie, Yishan Chen, Ansheng Xie, Shuheng Li, Leiqiong Gao, and Zhiying Yu interpreted the results of the experiments. Lian Zhang, Weiwei Deng, Di Wang, and Bin Yang drafted the manuscript. Yunsheng Liang, Weiwei Deng, Lian Zhang, Yuan Chang, Haijing Wu, Yan Ding, and Yingping Xu edited and revised the manuscript. Di Wang, Yuan Chang, Lian Zhang, and Yunsheng Liang approved the final version of the manuscript.

## Conflicts of interest

The authors declare that they have no known competing financial interests or personal relationships that could have appeared to influence the work reported in this paper.

## Appendix A Supporting information

The following is the Supporting Information to this article:

## References

[1] Delhommeau F., Dupont S., Della Valle V., James C., Trannoy S., Massé A. (2009). Mutation in TET2 in myeloid cancers. N Engl J Med.

[2] Xu Y.P., Lv L., Liu Y., Smith M.D., Li W.C., Tan X.M. (2019). Tumor suppressor TET2 promotes cancer immunity and immunotherapy efficacy. J Clin Investig.

[3] Pastor W.A., Aravind L., Rao A. (2013). TETonic shift: biological roles of TET proteins in DNA demethylation and transcription. Nat Rev Mol Cell Biol.

[4] Lian C.G., Xu Y., Ceol C., Wu F., Larson A., Dresser K. (2012). Loss of 5-hydroxymethylcytosine is an epigenetic hallmark of melanoma. Cell.

[5] Blaschke K., Ebata K.T., Karimi M.M., Zepeda-Martínez J.A., Goyal P., Mahapatra S. (2013). Vitamin C induces Tet-dependent DNA demethylation and a blastocyst-like state in ES cells. Nature.

[6] Liu M., Ohtani H., Zhou W., Ørskov A.D., Charlet J., Zhang Y.W. (2016). Vitamin C increases viral mimicry induced by 5-aza-2’-deoxycytidine. Proc Natl Acad Sci U S A.

[7] Lee M., Li J., Li J., Fang S., Zhang J., Vo A.T.T. (2021). Tet2 inactivation enhances the antitumor activity of tumor-infiltrating lymphocytes. Cancer Res.

[8] Pandey S.P., Bender M.J., McPherson A.C., Phelps C.M., Sanchez L.M., Rana M. (2022). Tet2 deficiency drives liver microbiome dysbiosis triggering Tc1 cell autoimmune hepatitis. Cell Host Microbe.

[9] Tsagaratou A., González-Avalos E., Rautio S., Scott-Browne J.P., Togher S., Pastor W.A. (2017). TET proteins regulate the lineage specification and TCR-mediated expansion of iNKT cells. Nat Immunol.

[10] Yang R., Qu C., Zhou Y., Konkel J.E., Shi S., Liu Y. (2015). Hydrogen sulfide promotes Tet1- and Tet2-Mediated Foxp3 demethylation to drive regulatory T cell differentiation and maintain immune homeostasis. Immunity.

[11] Jankowska A.M., Szpurka H., Tiu R.V., Makishima H., Afable M., Huh J. (2009). Loss of heterozygosity 4q24 and TET2 mutations associated with myelodysplastic/myeloproliferative neoplasms. Blood.

[12] Pan W., Zhu S., Qu K., Meeth K., Cheng J., He K. (2017). The DNA methylcytosine dioxygenase Tet2 sustains immunosuppressive function of tumor-infiltrating Myeloid cells to promote melanoma progression. Immunity.

[13] Thienpont B., Steinbacher J., Zhao H., D’Anna F., Kuchnio A., Ploumakis A. (2016). Tumour hypoxia causes DNA hypermethylation by reducing TET activity. Nature.

[14] Wu D., Hu D., Chen H., Shi G., Fetahu I.S., Wu F. (2018). Glucose-regulated phosphorylation of TET2 by AMPK reveals a pathway linking diabetes to cancer. Nature.

[15] Zhang Y.W., Wang Z., Xie W., Cai Y., Xia L., Easwaran H. (2017). Acetylation enhances TET2 function in protecting against abnormal DNA methylation during oxidative stress. Mol Cell.

[16] Nakagawa T., Lv L., Nakagawa M., Yu Y., Yu C., D’Alessio A.C. (2015). CRL4(VprBP) E3 ligase promotes monoubiquitylation and chromatin binding of TET dioxygenases. Mol Cell.

[17] Peng Y.J., Makarenko V.V., Nanduri J., Vasavda C., Raghuraman G., Yuan G. (2014). Inherent variations in CO-H2S-mediated carotid body O2 sensing mediate hypertension and pulmonary edema. Proc Natl Acad Sci U S A.

[18] Szabo C., Coletta C., Chao C., Módis K., Szczesny B., Papapetropoulos A. (2013). Tumor-derived hydrogen sulfide, produced by cystathionine-*β*-synthase, stimulates bioenergetics, cell proliferation, and angiogenesis in colon cancer. Proc Natl Acad Sci U S A.

[19] Ou Q., Qiao X., Li Z., Niu L., Lei F., Cheng R. (2024). Apoptosis releases hydrogen sulfide to inhibit Th17 cell differentiation. Cell Metab.

[20] Wang D., Li S., Chen Y., Luo J., Li L., Wang B. (2023). Sodium thiosulfate inhibits epithelial-mesenchymal transition in melanoma *via* regulating the Wnt/β-catenin signaling pathway. J Dermatol Sci.

[21] Wang D., Liu Y., Zong X., Li X., Yang S., Zeng Y. (2023). Sodium thiosulfate ameliorates atopic dermatitis *via* inhibiting the activation of NLRP3 inflammasome. Biochem Biophys Res Commun.

[22] Chen C.H., Huang C.Y., Lin H.H., Wang M.C., Chang C.Y., Cheng Y.F. (2021). Association of sodium thiosulfate with risk of ototoxic effects from platinum-based chemotherapy: a systematic review and meta-analysis. JAMA Netw Open.

[23] Wen W., Portales-Castillo I., Seethapathy R., Durant O., Mengesha B., Krinsky S. (2023). Intravenous sodium thiosulphate for Calciphylaxis of chronic kidney disease: a systematic review and meta-analysis. JAMA Netw Open.

[24] Sen N., Paul B.D., Gadalla M.M., Mustafa A.K., Sen T., Xu R. (2012). Hydrogen sulfide-linked sulfhydration of NF-κB mediates its antiapoptotic actions. Mol Cell.

[25] Filipovic M.R., Zivanovic J., Alvarez B., Banerjee R. (2018). Chemical biology of H_2_S signaling through persulfidation. Chem Rev.

[26] Iciek M., Kowalczyk-Pachel D., Bilska-Wilkosz A., Kwiecień I., Górny M., Włodek L. (2015). S-sulfhydration as a cellular redox regulation. Biosci Rep.

[27] Ida T., Sawa T., Ihara H., Tsuchiya Y., Watanabe Y., Kumagai Y. (2014). Reactive cysteine persulfides and S-polythiolation regulate oxidative stress and redox signaling. Proc Natl Acad Sci U S A.

[28] Paul B.D., Snyder S.H. (2012). H_2_S signalling through protein sulfhydration and beyond. Nat Rev Mol Cell Biol.

[29] Panagaki T., Randi E.B., Augsburger F., Szabo C. (2019). Overproduction of H(2)S, generated by CBS, inhibits mitochondrial Complex IV and suppresses oxidative phosphorylation in Down syndrome. Proc Natl Acad Sci U S A.

[30] Kosower N.S., Kosower E.M., Wertheim B., Correa W.S. (1969). Diamide, a new reagent for the intracellular oxidation of glutathione to the disulfide. Biochem Biophys Res Commun.

[31] Schneider-Poetsch T., Ju J., Eyler D.E., Dang Y., Bhat S., Merrick W.C. (2010). Inhibition of eukaryotic translation elongation by cycloheximide and lactimidomycin. Nat Chem Biol.

[32] Liu Y., Yang R., Liu X., Zhou Y., Qu C., Kikuiri T. (2014). Hydrogen sulfide maintains mesenchymal stem cell function and bone homeostasis *via* regulation of Ca(2+) channel sulfhydration. Cell Stem Cell.

[33] Hu L., Li Z., Cheng J., Rao Q., Gong W., Liu M. (2013). Crystal structure of TET2-DNA complex: insight into TET-mediated 5mC oxidation. Cell.

[34] Sun X., Zhou X., Du L., Liu W., Liu Y., Hudson L.G. (2014). Arsenite binding-induced zinc loss from PARP-1 is equivalent to zinc deficiency in reducing PARP-1 activity, leading to inhibition of DNA repair. Toxicol Appl Pharmacol.

[35] Ribas A., Dummer R., Puzanov I., VanderWalde A., Andtbacka R.H.I., Michielin O. (2017). Oncolytic virotherapy promotes intratumoral T cell infiltration and improves Anti-PD-1 immunotherapy. Cell.

[36] Peng D., Kryczek I., Nagarsheth N., Zhao L., Wei S., Wang W. (2015). Epigenetic silencing of TH1-type chemokines shapes tumour immunity and immunotherapy. Nature.

[37] Lee J.H., Shklovskaya E., Lim S.Y., Carlino M.S., Menzies A.M., Stewart A. (2020). Transcriptional downregulation of MHC class I and melanoma de- differentiation in resistance to PD-1 inhibition. Nat Commun.

[38] Ivashkiv L.B., Donlin L.T. (2014). Regulation of type I interferon responses. Nat Rev Immunol.

[39] Liu Y., Liu P., Yang C., Cowley A.W., Liang M. (2014). Base-resolution maps of 5-methylcytosine and 5-hydroxymethylcytosine in Dahl S rats: effect of salt and genomic sequence. Hypertension.

[40] Alspach E., Lussier D.M., Schreiber R.D. (2019). Interferon γ and its important roles in promoting and inhibiting spontaneous and therapeutic cancer immunity. Cold Spring Harbor Perspect Biol.

[41] Saldanha G., Joshi K., Lawes K., Bamford M., Moosa F., Teo K.W. (2017). 5-Hydroxymethylcytosine is an independent predictor of survival in malignant melanoma. Mod Pathol.

[42] Gao X.H., Krokowski D., Guan B.J., Bederman I., Majumder M., Parisien M. (2015). Quantitative H2S-mediated protein sulfhydration reveals metabolic reprogramming during the integrated stress response. eLife.

[43] Mustafa A.K., Gadalla M.M., Sen N., Kim S., Mu W., Gazi S.K. (2009). H2S signals through protein S-sulfhydration. Sci Signal.

[44] Guo C., You Z., Shi H., Sun Y., Du X., Palacios G. (2023). SLC38A2 and glutamine signalling in cDC1s dictate anti-tumour immunity. Nature.

[45] Singh P., Gollapalli K., Mangiola S., Schranner D., Yusuf M.A., Chamoli M. (2023). Taurine deficiency as a driver of aging. Science.

[46] Fang L., Hao Y., Yu H., Gu X., Peng Q., Zhuo H. (2023). Methionine restriction promotes cGAS activation and chromatin untethering through demethylation to enhance antitumor immunity. Cancer Cell.

[47] Huang A.C., Zappasodi R. (2022). A decade of checkpoint blockade immunotherapy in melanoma: understanding the molecular basis for immune sensitivity and resistance. Nat Immunol.

[48] Lim S.Y., Shklovskaya E., Lee J.H., Pedersen B., Stewart A., Ming Z. (2023). The molecular and functional landscape of resistance to immune checkpoint blockade in melanoma. Nat Commun.

[49] Schoenfeld A.J., Hellmann M.D. (2020). Acquired resistance to immune checkpoint inhibitors. Cancer Cell.

