# Supplementary material for: Loss of TET2 sulfhydration results in destabilization of TET2 and down-regulation of the immune response in melanoma
Source: Acta Pharm Sin B. 2026 Apr 19;16(8):5183–99. doi: 10.1016/j.apsb.2026.04.010 (PMC13464072; doi:10.1016/j.apsb.2026.04.010)
Supplement: Supplementary file 1 [file mmc1.pdf]

Supporting Information for

Original article

Loss of TET2 sulfhydration results in destabilization of TET2 and down-regulation of the immune response in melanoma

Di Wang[b,†], Weiwei Deng[b,†], Yang Xie[c,†], Ansheng Xie[a,b,†], Yuan Chang[d], Shuheng Li[b], Yishan Chen[b], Leiqiong Gao[e], Yan Zhang[b], Zhiying Yu[b], Bocheng Wang[b], Yingping Xu[b], Haijing Wu[a], Yan Ding[f], Bin Yang[b], Lian Zhang[b,*], Yunsheng Liang[a,b,*]

[a]*Hunan Key Laboratory of Medical Epigenomics & Department of Dermatology, The Second Xiangya Hospital of Central South University, Changsha 410011, China*

[b]*Dermatology Hospital of Southern Medical University, Guangzhou 510091, China*

[c]*Department of Dermatology, The 3rd Affiliated Hospital of Sun Yat-sen University, Guangzhou 510630, China*

[d]*Department of Neurology, Nanfang Hospital, Southern Medical University, Guangzhou 510515, China*

[e]*Institute of Immunological Innovation and Translation, Chongqing Medical University, Chongqing 400016, China*

[f]*Department of Dermatology, Hainan Provincial Hospital of Skin Disease, Haikou 570206, China*

Received 16 August 2025; received in revised form 8 October 2025; accepted 3 November 2025

*Corresponding authors.

E-mail addresses: yunshengliang@aliyun.com (Yunsheng Liang), 907189585@qq.com (Lian Zhang).

[†]These authors made equal contributions to this work.

1. Supporting figures

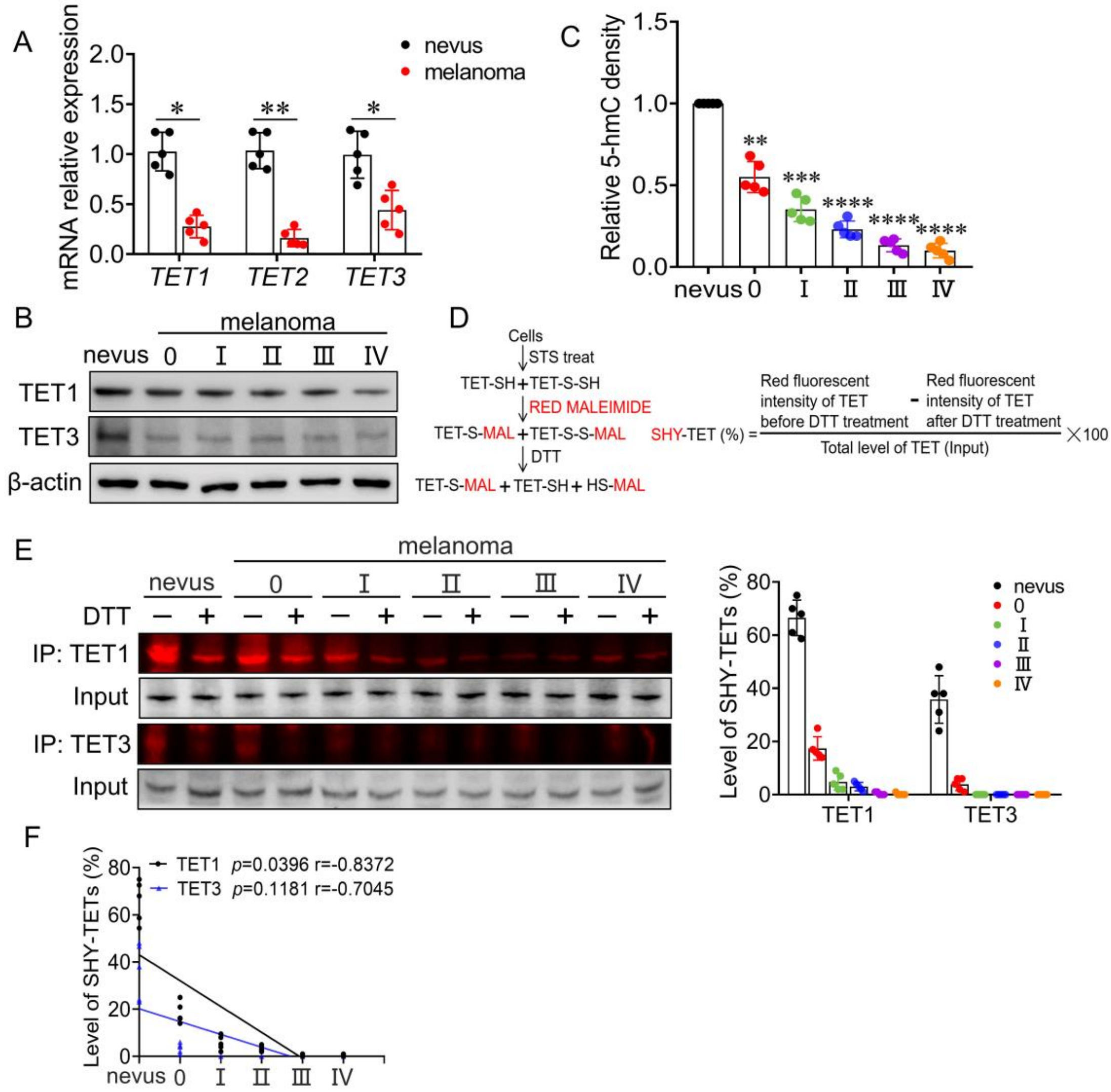

Figure S1 The expression and sulfhydration levels of TET1 and TET3 proteins in melanoma patients.

(A) TET mRNA expression levels in patients with nevus and melanoma. (B) TET1 and TET3 protein expression levels in patients with nevus (*n* = 5) and melanoma (*n* = 25) at different stages. (C) Dynamic changes in 5-hmC expression levels along with melanoma pathological stage. (D) Schematic diagram for the detection of TET sulfhydration with red maleimide. (E) TET1 and TET3 sulfhydration levels in patients with nevus (*n* = 5) or melanoma (*n* = 25). (F) Analysis of the correlation between TET1 and TET3 protein sulfhydration levels and the clinical stages of melanoma. Statistical analyses were performed using one-way ANOVA followed by Tukey's multiple comparisons test for Panels C and E and Spearman's correlation analysis for Panel F. Experiments were conducted in triplicate. $*P < 0.05$, $**P < 0.01$, $***P < 0.001$, $****P < 0.0001$.

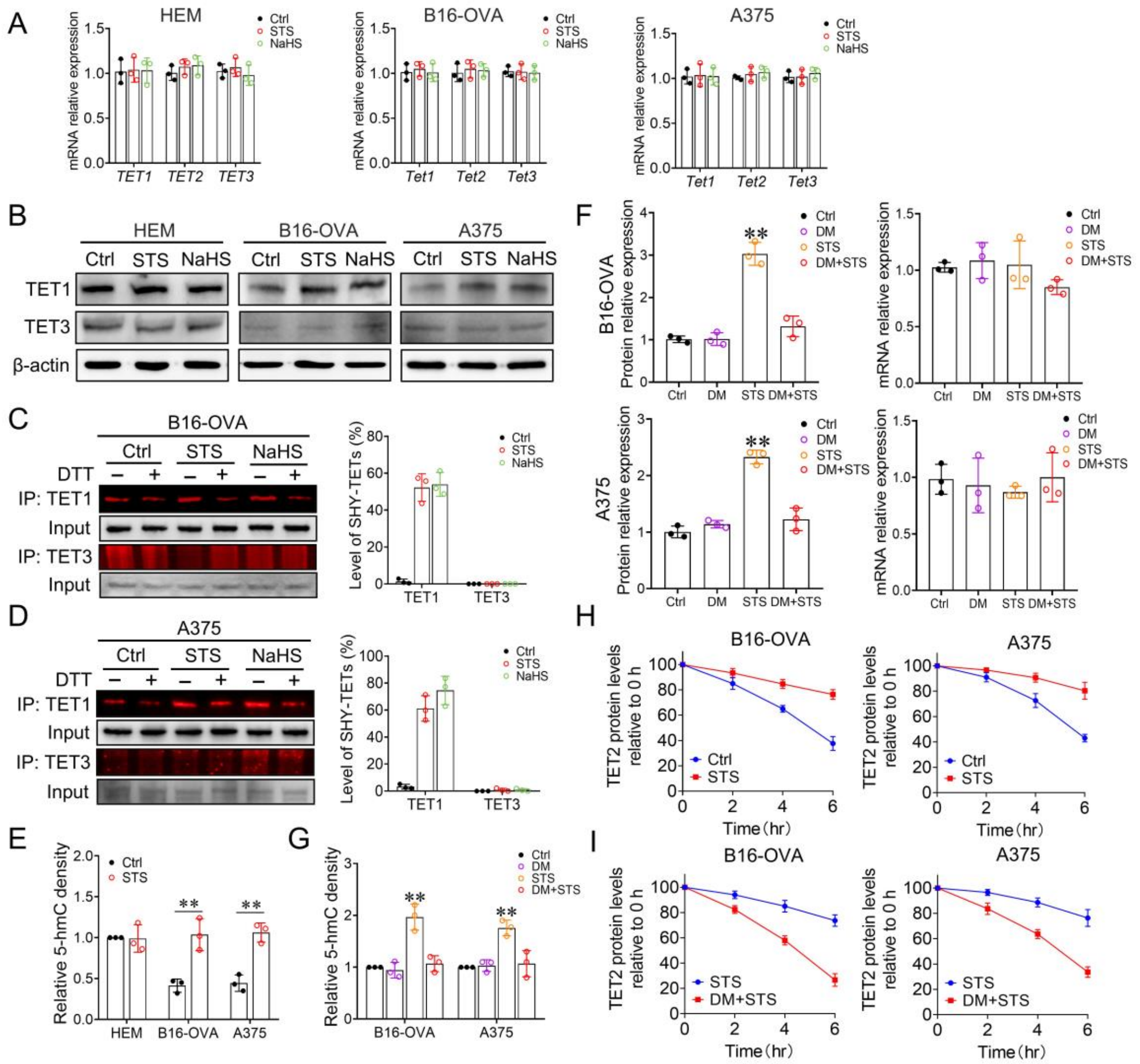

Figure S2 Active sulfur restores the expression and catalytic activity of the TET protein. (A) TET mRNA expression levels in melanocytes and melanoma cell lines treated with active sulfur. (B–D) TET1 and TET3 protein expression (B) and sulfhydration levels (C, D) in melanocytes and melanoma cell lines treated with active sulfur. (E) Global 5-hmC expression levels in melanoma cell lines after active sulfur treatment. (F) TET2 protein and mRNA expression levels in melanoma cell lines after treatment with active sulfur combined with or without the sulfhydration inhibitor DM. (G) Global 5-hmC expression levels in melanoma cell lines after treatment with active sulfur and sulfhydration inhibitors. (H) TET2 expression levels in melanoma cell lines at different time points after active sulfur treatment. (I) TET2 protein expression in melanoma cells after the indicated treatments. Statistical significance was assessed by one-way ANOVA across three independent experiments, and the data are presented as the mean ± SD. n = 3. Three independent experiments were performed. **$P < 0.01$.

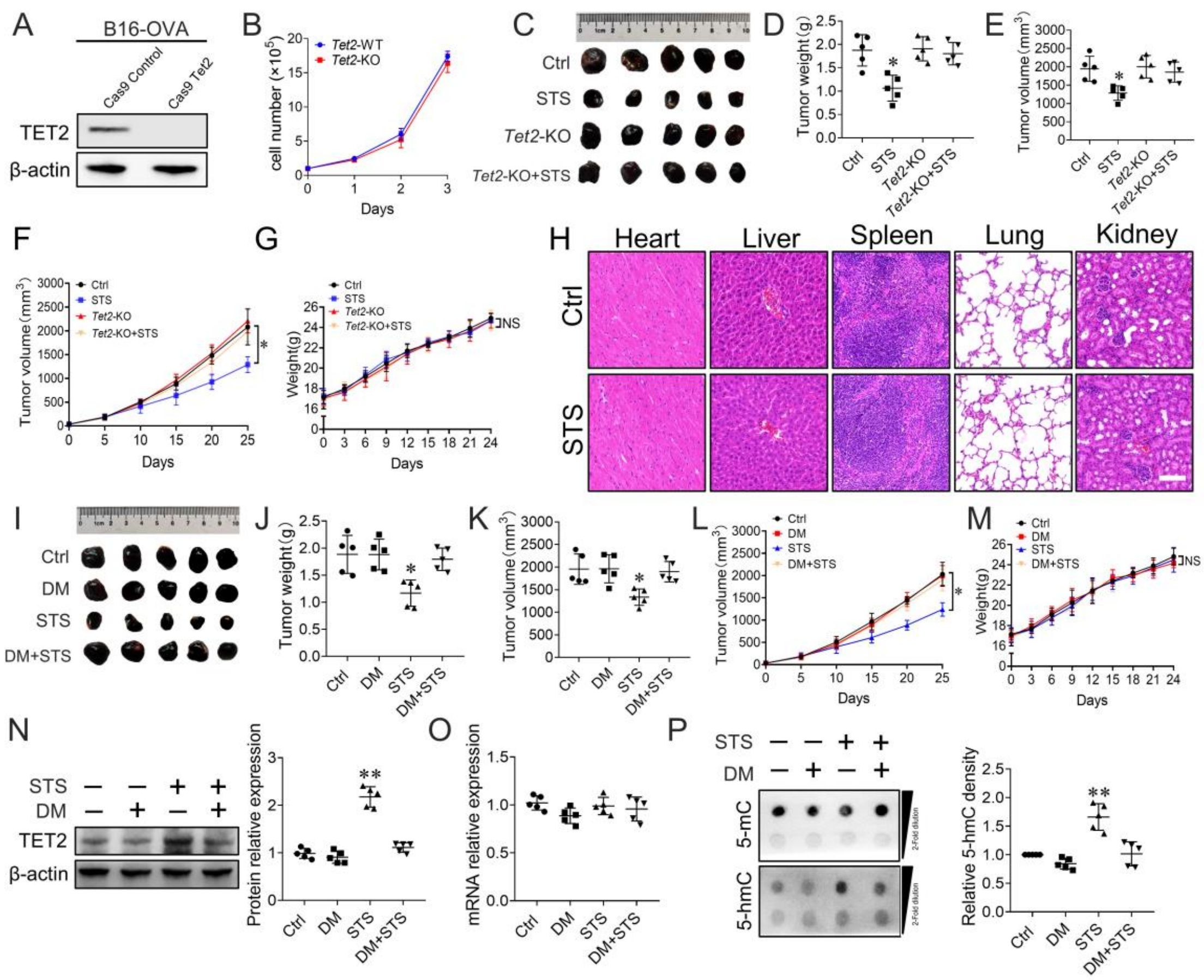

Figure S3 The antimelanoma effect of active sulfur depends on the sulfhydration of the TET2 protein.

(A) TET2 protein expression level after CRISPR-Cas9-mediated knockout of *Tet2* in the B16-OVA cell line determined by Western blotting. (B) Proliferation rates of *Tet2*-WT B16-OVA and *Tet2*-KO B16-OVA cells cultured *in vitro*. (C–G) Tumor volume, body weight and tumor size of the *Tet2*-WT and *Tet2*-KO melanoma tumor-bearing mice in the indicated groups. (H) H&E staining results of various organs between the two groups. (I–M) Tumor volume, body weight and tumor size in the indicated groups of melanoma tumor-bearing mice after treatment with active sulfur and the sulfhydration inhibitor DM. (N–P) TET2 protein (N) and mRNA (O) and 5-mC and 5-hmC levels (P) in tumor tissue after treatment with active sulfur and the sulfhydration inhibitor DM. The data are presented as the mean ± SD. Statistical significance was assessed by one-way ANOVA followed by Tukey's multiple comparisons test. $n = 5$. Three independent experiments were performed. *$P < 0.05$, **$P < 0.01$; NS indicates not significant.

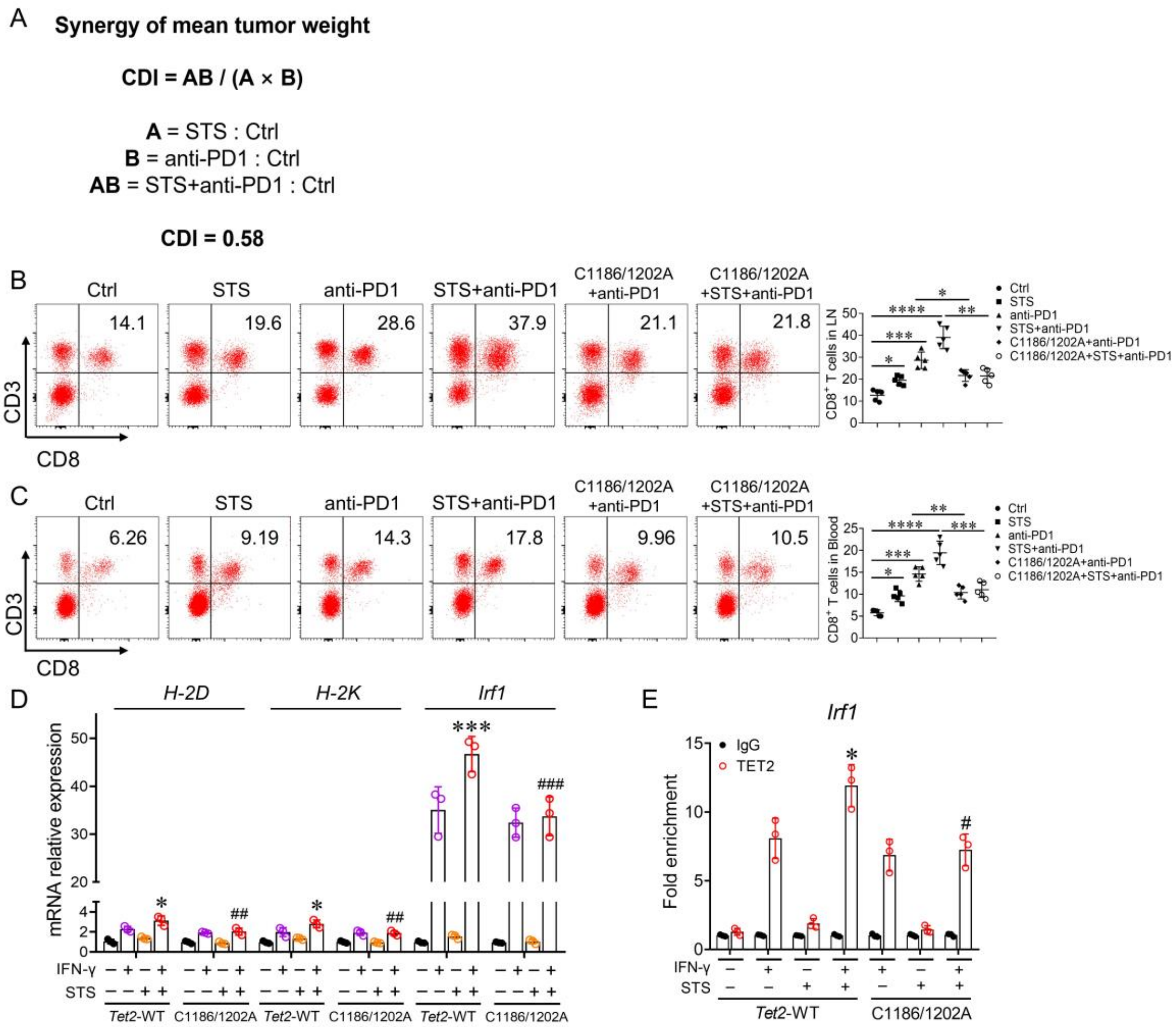

Figure S4 Active sulfur synergizes with anti-PD-1 antibodies to promote the accumulation of CD8$^+$ T cells.

(A) Synergistic index calculation showing that the combined group has synergistic effects. (B, C) Proportion of CD8$^+$ T lymphocytes in the lymph nodes (B) and blood (C) of the mice in the indicated groups. The data are presented as the mean ± SD (n = 5). (D) mRNA expression levels of *H-2D*, *H-2K*, and *Irf1* in *Tet2*-WT and mutant cells (n = 5). (E) ChIP assay assessing TET2 enrichment at the *Irf1* promoter in the indicated groups (n = 3). Statistical significance was assessed by one-way ANOVA followed by Tukey's multiple comparisons test. Three independent experiments were performed. *P < 0.05, **P < 0.01, ***P < 0.001, ****P < 0.0001.

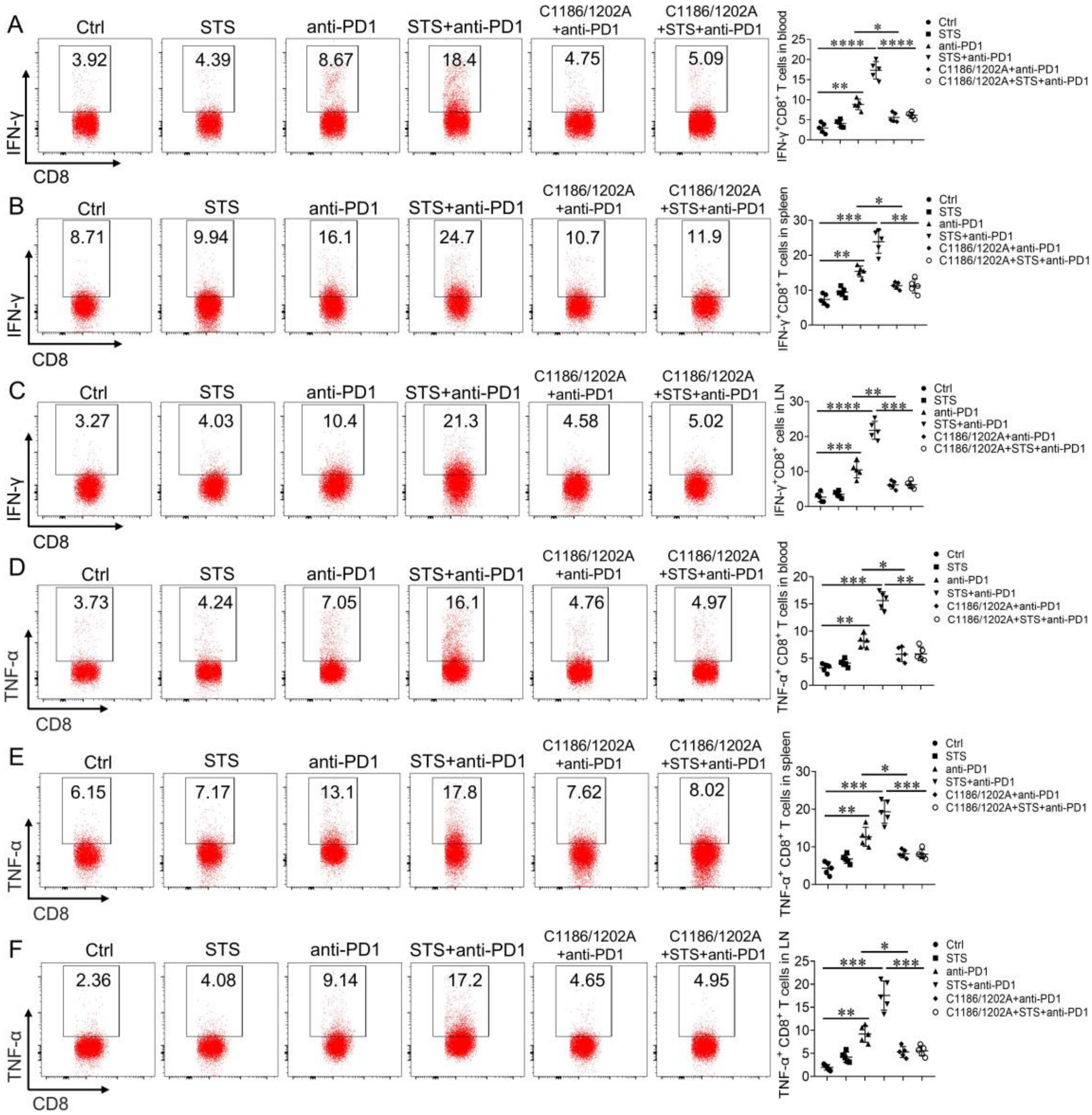

Figure S5 Active sulfur combined with anti-PD-1 antibodies enhances IFN-γ and TNF-α expression.

(A–C) Proportion of IFN-γ^{+}CD8^{+} cells in different tissues, including blood (A), spleen (B), and lymph nodes (C) in the indicated groups. (D–F) Proportion of TNF-α^{+}CD8^{+} cells in different tissues, including blood (D), spleen (E), and lymph nodes (F) in the indicated groups. Statistical significance was assessed by one-way ANOVA followed by Tukey's multiple comparisons test. Three independent experiments were performed. *$P < 0.05$, **$P < 0.01$, ***$P < 0.001$, ****$P < 0.0001$.

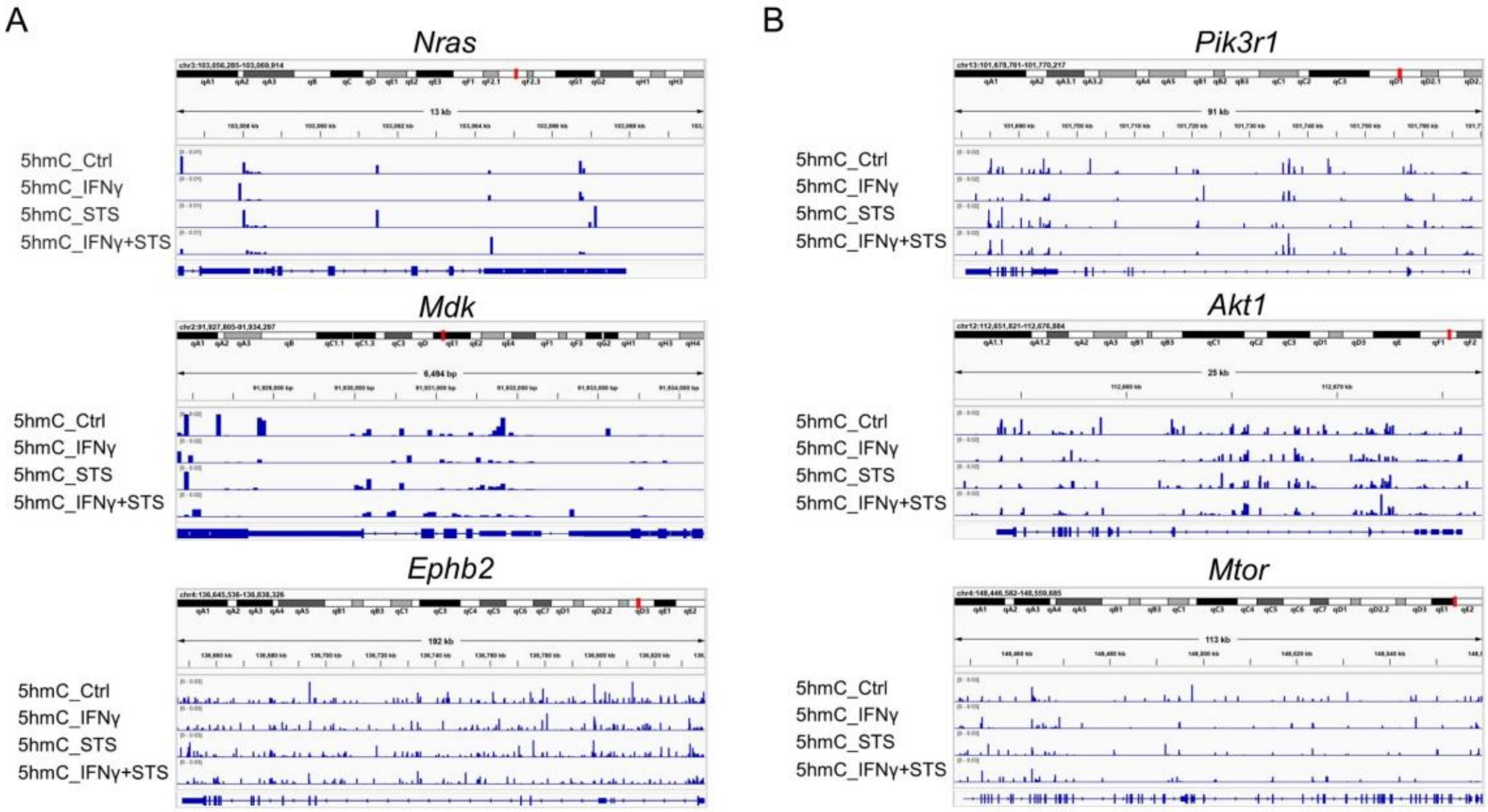

Figure S6 Distribution of 5-hmC densities in the key oncogenes of melanoma.
